# Structural Insights into *Escherichia coli* Shiga Toxin (Stx) Glycosphingolipid Receptors of Porcine Renal Epithelial Cells and Inhibition of Stx-Mediated Cellular Injury Using Neoglycolipid-Spiked Glycovesicles

**DOI:** 10.3390/microorganisms7110582

**Published:** 2019-11-19

**Authors:** Johanna Detzner, Caroline Gloerfeld, Gottfried Pohlentz, Nadine Legros, Hans-Ulrich Humpf, Alexander Mellmann, Helge Karch, Johannes Müthing

**Affiliations:** 1 Institute for Hygiene, University of Münster, 48149 Münster, Germany; Johanna.Detzner@ukmuenster.de (J.D.); caroline.gloerfeld@web.de (C.G.); pohlentz@uni-muenster.de (G.P.); nadine.legros@me.com (N.L.); Alexander.Mellmann@ukmuenster.de (A.M.); Helge.Karch@ukmuenster.de (H.K.); 2 Institute for Food Chemistry, University of Münster, 48149 Münster, Germany; humpf@uni-muenster.de

**Keywords:** edema disease, epithelial cells, Gb3Cer, Gb4Cer, glycosphingolipids, LLC-PK1, neoglycolipids, PK-15, porcine kidney, Stx2e

## Abstract

Shiga toxin (Stx) producing *Escherichia coli* (STEC) cause the edema disease in pigs by releasing the swine-pathogenic Stx2e subtype as the key virulence factor. Stx2e targets endothelial cells of animal organs including the kidney harboring the Stx receptor glycosphingolipids (GSLs) globotriaosylceramide (Gb3Cer, Galα1-4Galβ1-4Glcβ1-1Cer) and globotetraosylceramide (Gb4Cer, GalNAcβ1-3Galα1-4Galβ1-4Glcβ1-1Cer). Since the involvement of renal epithelial cells in the edema disease is unknown, in this study, we analyzed the porcine kidney epithelial cell lines, LLC-PK1 and PK-15, regarding the presence of Stx-binding GSLs, their sensitivity towards Stx2e, and the inhibitory potential of Gb3- and Gb4-neoglycolipids, carrying phosphatidylethanolamine (PE) as the lipid anchor, towards Stx2e. Immunochemical and mass spectrometric analysis revealed various Gb3Cer and Gb4Cer lipoforms as the dominant Stx-binding GSLs in both LLC-PK1 and PK-15 cells. A dihexosylceramide with proposed Galα1-4Gal-sequence (Gal_2_Cer) was detected in PK-15 cells, whereas LLC-PK1 cells lacked this compound. Both cell lines were susceptible towards Stx2e with LLC-PK1 representing an extremely Stx2e-sensitive cell line. Gb3-PE and Gb4-PE applied as glycovesicles significantly reduced the cytotoxic activity of Stx2e towards LLC-PK1 cells, whereas only Gb4-PE exhibited some protection against Stx2e for PK-15 cells. This is the first report identifying Stx2e receptors of porcine kidney epithelial cells and providing first data on their Stx2e-mediated damage suggesting possible involvement in the edema disease.

## 1. Introduction

Edema disease usually occurs in pigs shortly after weaning and exhibits typical clinical neurologic signs such as ataxia, convulsions, and paralysis, or causes sudden death [1,2]. The disease was named “edema disease” or “gut edema” because primordial investigations of veterinary researchers revealed excess fluid in the walls of the stomach and intestine or under the eyelids of affected pigs. However, involvement of the brain is crucial and causes the clinical symptoms [3,4]. Porcine edema disease is an enterotoxemia caused by certain pathogenic strains of *Escherichia coli* that colonize the small intestine and produce Shiga toxin (Stx) of the Stx2e subtype considered the key virulence factor involved in the pathogenesis of the infection [3,4]. F18ab fimbriae mediate bacterial colonization, while Stx2e upon transfer to the circulation injures brain endothelial cells, ranging from acute swelling to necrosis and detachment from basement membrane, as an early event in the pathogenesis of Stx-producing *E. coli* (STEC) strains [5]. Damage of the blood vessels has an effect on blood pressure and causes leakage of fluid from vessels resulting in accumulation in a number of body tissues. The Stx2e-mediated breakdown of the blood-brain barrier has been shown employing an in vitro model monitoring the collapse of the transendothelial electrical resistance of porcine brain endothelial cells in real time [6,7]. Moreover, the edema disease of swine has been used as a model to study the pathogenesis of similar diseases of human beings due to comparative pathology that manifests as edema disease in swine and hemolytic uremic syndrome (HUS) in humans caused by enterohemorrhagic *E. coli* (EHEC) that represent the human-pathogenic STEC subgroup [8]. Despite the low frequency of Stx2e-producing STEC among human clinical isolates and their general association with a mild course of infections [9,10,11], Stx2e-producing *E. coli* strains have also been occasionally isolated from humans with HUS [12,13]. However, the relationship between swine STEC and human disease requires further evaluation [14,15,16,17,18].

Early studies have shown the attachment of Stx2e [named as VT2e, SLT-IIv or SLT-IIe at that time [19,20,21,22] to various tissues of the gastrointestinal tract (stomach, colon, small intestine, and duodenum) and other organs including the kidney of weanling piglets [23,24,25]. Previously unreported Stx binding sites were identified in porcine kidney tubules [26], and kidney lesions, similar to those in humans with HUS, were observed in piglets inoculated intragastrically with STEC O157:H7 [27]. The Stx receptor globotriaosylceramide (Gb3Cer, Galα1-4Galβ1-4Glcβ1-1Cer) was localized immunohistochemically at sites of the renal lesions that matched with the locations of Stx binding. The various lipoforms of Gb3Cer and globotetraosylceramide (Gb4Cer, GalNAcβ1-3Galα1-4Galβ1-4Glcβ1-1Cer), known as moderate and preferred glycosphingolipid (GSL) receptor of Stx2e, respectively [28,29,30], have been recently scrutinized in GSL preparations of porcine cortex, medulla, and pelvis of a male and a female piglet [31]. The dominant variants of Gb3Cer and Gb4Cer were identified immunochemically by thin-layer chromatography (TLC) overlay detection combined with electrospray ionization mass spectrometry (ESI MS). Structural analysis has revealed Gb3Cer and Gb4Cer lipoforms that exhibited an almost balanced profile of species carrying sphingosine (d18:1) as the constant portion and variable fatty acids with chain lengths from C16 to C24 in the various organs [31]. In striking contrast to Stx1a and Stx2a, Stx2e binds to the extended globo-series GSLs globopentaosylceramide (Gb5Cer, Galβ1-3 GalNAcβ1-3Galα1-4Galβ1-4Glcβ1-1Cer), corresponding to Gb4Cer extended by a galactose (Gal) in β1-3-configuration [32] and Forssman GSL, corresponding to Gb4Cer elongated by an *N*-acetylgalactosamine (GalNAc) in α1-3-configuration [30]. This feature makes Stx2e unique among the various Stx subtypes (for revised nomenclature of Stxs refer to [33]) analyzed in detail so far, although the biological significance remains obscure.

Stx-mediated damage of the human kidney throughout the manifestation of HUS is primarily due to damage of glomerular endothelial cells [34,35,36,37,38,39,40], however, evidence has accumulated for involvement of renal epithelia cells in kidney injury. Numerous in vitro studies have shown the cytotoxic action of the human-pathogenic Stx1a and Stx2a subtypes (in previous publications denoted as Stx1 and Stx2, respectively) towards primary human glomerular and tubular epithelial cells of the kidney [41,42,43,44,45,46,47,48]. In addition, kidney-derived epithelial cell lines have been shown to be susceptible for Stx, such as the ACHN cell line, which is an in vitro model of renal tubular epithelial cells [49,50], and HK-2, a human proximal tubule cell line [51,52,53,54]. In mice, renal tubular epithelial cells have expressed the Stx receptor Gb3Cer and responded to Stx2 [55,56,57,58]. Furthermore, Stx has been found to target the murine renal collecting duct epithelium in vivo, and thereby, contribute to renal failure [55] and cause pathological changes of the kidney similar to those seen in humans with HUS [56]. Last but not least, application of Stx has elicited injury of the renal tubular epithelium in mice and revealed, in addition to the protective physiologic role of Gb3Cer [57], a pathophysiologic role of Gb3Cer in the proximal tubules [58].

The renal cell function of the pig has been studied using the LLC-PK1 cell line, which exhibits properties characteristic of swine kidney proximal tubular epithelium [59,60]. PK-15 is another porcine cell line exhibiting kidney epithelial features often used for studying the molecular mechanisms of porcine virus infection or virus production [61,62,63,64]. Regarding the involvement in edema disease, the presence of Stx receptors and their possible interaction with Stxs are completely unknown for LLC-PK1 and PK-15 cells. The aims of this study were the identification and structural characterization of assumed Stx-binding GSLs of the two porcine kidney epithelial cell lines, LLC-PK1 and PK-15, the possible Stx2e-mediated damage of this porcine cell type, and the contingent inhibition of Stx2e-mediated cellular damage using neoglycolipid-spiked glycovesicles.

## 2. Materials and Methods

### 2.1. Cultivation of LLC-PK1 and PK-15 Cells

The pig LLC-PK1 cell line (No. ACC 637) and the pig PK-15 cell line (No. ACC 640) were obtained from the German Collection of Microorganisms and Cell Cultures GmbH (DSMZ, Braunschweig, Germany). The pig LLC-PK1 cell line was established from the normal kidney of a juvenile Hampshire pig (*Sus scrofa*) and exhibits properties characteristic of kidney proximal tubular epithelium [59,65,66]. PK-15 is a homogeneous epithelial cell line from porcine kidney, which was originally established from the PK-2a cell line [67] and has found wide application in analyzing the replicate mechanism of porcine virus infection [68,69]. Light microscopy micrographs taken from porcine LLC-PK1 and PK-15 cells are shown in Supplementary Figure S1A,B, respectively, demonstrating the characteristic epithelial phenotype of the utilized renal cell lines. The cells were propagated under serum-free conditions in OptiPRO^TM^SFM (Gibco Life Technologies Corporation, Paisley, UK) supplemented with 4 mM L-glutamine in a humidified atmosphere with 5% CO_2_ at 37 °C, as previously described by [70]. The LLC-PK1 and PK-15 cells were passaged in a split ratio of 1:10 and 1:5, respectively, each in 3-day intervals. Adequate cell masses for the isolation of GSLs were produced according to earlier published specifications [71].

### 2.2. Preparation of the Neoglycolipids Gb3-PE and Gb4-PE

Globotriaose (Gb3, Galα1-4Galβ1-4Glc; ELICITYL, Crolles, France, GLY120, Lot E1110-05 EC05, 98% purity), globotetraose (Gb4, GalNAcβ1-3Galα1-4Galβ1-4Glc; ELICITYL, GLY121, Lot E1501-11 EC04 > 90% purity) and phospatidylethanolamine (PE, 1,2-dihexadecyl-*sn*-glycero-3-phosphoethanolamine; Merck, Darmstadt, Germany, no. 37161 (> 99% purity) were used for the synthesis of the neoglycolipids Gb3-PE and GB4-PE following a previously published protocol [72]. All other reagents and solvents were of highest purity available. For the synthesis of Gb3-PE, the amount of 101 mg of Gb3 (200.4 µmol) was taken up in 500 µL of dimethyl sulfoxide (DMSO) and diluted with 3.5 mL of methanol. A molar 1.2 equivalent of PE corresponding to 159.6 mg (240.6 µmol) dissolved at 10 mg/mL in chloroform/methanol (2/1, *v/v*) was added, and the mixture was incubated at 60 °C for 2 h. Subsequently, 40 mg of NaBH_3_CN dispensed at 10 mg/mL in chloroform/methanol (1/1, *v/v*) containing 1% acetic acid was successively added in 8 steps of 500 µL each over a period of 20 h while keeping the reaction mixture at 60 °C. Afterwards the solvent was thoroughly evaporated under a stream of nitrogen. The residue was suspended in 50 mL of water and the suspension was dialyzed against 5 L of water for 48 h with a three-fold water exchange. The retentate was lyophilized and yielded 184.5 mg of Gb3-PE (79.9% relative to the amount of Gb3). Gb4-PE was synthesized according to this procedure. The amount of 20.3 mg of Gb4 (28.7 µmol) dispersed in 100 µL of DMSO was diluted in 750 µL of methanol, mixed with 23.8 mg (35.8 µmol) of PE in chloroform/methanol (2/1, *v/v*), and heated to 60 °C for 2 h. The NaBH_3_CN solution was applied in 6 portions of 200 µL each. Evaporation, dialysis, and lyophilization were performed, as described for the synthesis of Gb3-PE above, and yielded 27.3 mg of Gb4-PE (70.3% relative to the amount of Gb4).

### 2.3. Production of Neoglycolipid- and Gb3Cer-Loaded Glycovesicles

For the preparation of glycovesicles, the lipid compounds were merged in chloroform/methanol (2/1, *v/v*), evaporated, and resolved in phosphate-buffered saline (PBS), as previously described [73]. In short, solutions containing 0.4 mg of 1,2-dioleoyl-*sn*-glycero-3-phosphatidylcholine (DOPC, P6354) and 0.2 mg of cholesterol (C8667), both from Sigma-Aldrich (Steinheim, Germany), and 0.4 mg of synthesized Gb3-PE or Gb4-PE or reference Gb3Cer, each dissolved in chloroform/methanol (2/1, *v/v*), were thoroughly mixed. After evaporation of the organic solvents and hydration in 1 mL of PBS, the suspension was extruded through a polycarbonate membrane with 100 nm pore size yielding small unilamellar glycovesicles loaded with Gb3-PE or Gb4-PE or Gb3Cer [73].

### 2.4. Stx2e Cytotoxicity and Stx2e Inhibition Assay

Sterile filtrated Stx2e-containing supernatants were obtained from liquid cultures of STEC of human and porcine origin (see below, 2.6. Stx1a, Stx2a, Stx2e and anti-Stx, anti-GSL, and secondary antibodies). STEC strains derived from human (h) and porcine (p) isolates were used for the production of Stx2e(h)- and Stx2e(p)-containing supernatants, respectively. Supernatants were employed for probing Stx2e-mediated cell damage of porcine epithelial cell cultures (see below in this paragraph) and used in thin-layer chromatography overlay assays (see below, 2.7. Thin-layer chromatography and overlay assay) for analyzing the toxins’ binding specificities, as described in a previous publication [30]. The crystal violet assay served for determining Stx2e-caused cell killing rates [70]. The capability of glycovesicles loaded with the neoglycolipids, Gb3-PE or Gb4-PE to inhibit the Stx2e-mediated cellular damage of the pig epithelial cells as compared with glycovesicles spiked with the gold standard Gb3Cer, was probed as outlined in a very recent publication [72]. The Stx2e(h) supernatant was used as 1:2^15^ and 1:2^6^ dilutions for LLC-PK1 and PK-15 cells, respectively, and the Stx2e(p) supernatant was employed as 1:2^7^ dilution for LLC-PK1 cells in the glycovesicle inhibition assays. In short, cells were seeded in a 96-well microtiter plate with 4 × 10^3^ cells in 100 µL of serum-free cell culture medium per well and bred for 24 h (37 °C, 5% CO_2_). Stx-containing bacterial supernatants were diluted in cell culture medium and applied in 100 µL volumes in parallel to 100 µL medium only (control) for 1 h. The solutions were replaced by 200 µL of fresh medium per well and incubated for 48 h. The cell viability was determined photometrically as previously described [70]. For probing inhibition of Stx2e-caused cytotoxicity, solutions of glycovesicles spiked with Gb3-PE or Gb4-PE or Gb3Cer gold standard (see Section 2.3. Production of neoglycolipid- and Gb3Cer-loaded glycovesicles) were diluted 1:2 with Stx-containing medium or medium only (control) and incubated for 1 h. Thereafter, 100 µL cell cultures were exposed to 100 µL aliquots of these solutions for 1 h. The applied amounts of Gb3-PE, Gb4-PE, and Gb3Cer corresponded to 20 µg/well, respectively, and were equivalent to a concentration of 100 µg/mL in the cell culture. After removal of the supernatants, each well was supplied with 200 µL of fresh medium for 48 h. Photometrically determined cell viabilities are presented as means ± standard deviations (SD) of four independent measurements and are depicted as percentage values in relation to untreated control cells, which correspond to 100% viability. R software Microsoft Excel 2010 (version 14.0.7237.5000, Microsoft, Redmond, WA, USA) was employed for statistical analysis of the data obtained in the inhibition assays. The disparity among the sample groups was tested using analysis of variance (ANOVA). Student’s t-test was used for pairwise comparison of the groups, where differences were considered significant at *p* < 0.01 or *p* < 0.001.

### 2.5. Isolation of Neutral GSLs from LLC-PK1 and PK-15 Cells

Neutral GSLs were isolated from lipid extracts of two independent biological replicates of confluently grown LLC-PK1 and PK-15 cells, respectively, as previously described [74]. Briefly, the first extraction step of the cell layers was performed with methanol, followed by thorough stepwise extraction using chloroform/methanol mixtures with an increasing chloroform content of (1/2, *v/v*), (1/1, *v/v*), and (2/1, *v/v*). The extracts were combined, rotary evaporated, followed by saponification of alkali-labile phospholipids and triglycerides. The fraction harbouring the neutral GSLs was prepared by means of anion-exchange chromatography using a DEAE-Sepharose CL-6B (GE Healthcare, Munich, Germany) as described in earlier times [75], finally taken up in chloroform/methanol (2/1, *v/v*) and stored at −20 °C.

### 2.6. Stx1a, Stx2a, Stx2e and Anti-Stx, Anti-GSL, and Secondary Antibodies

STEC wild-type strains from human isolates of serotype O145:H- (strain 2074/97, Stx1a) and O111:H- (strain 03-06016, Stx2a) were the source of supernatants containing Stx1a and Stx2a, respectively. STEC strains of human (h) origin of serotype ONT:H- (strain 2771/97) and of porcine (p) origin of serotype O139:K82 (strain S115G) were used for the production of Stx2e(h)- and Stx2e(p)-containing supernatants, respectively, as described in a previous publication [30]. We sequenced strains 2074/97, 03-06016, 2771/97, and S115G as described previously [76] and extracted the genes encoding the Stx subunits A and B using nucleotide similarity searches. For comparison, *stx* reference sequences were used from Scheutz et al. [33]. These Stx-variants, combined with anti-Stx1 and anti-Stx2 antibody, as well as polyclonal chicken anti-Gb3Cer and anti-Gb4Cer antibodies were used in solid-phase binding assays (see below Section 2.7. Thin-layer chromatography and overlay assay) for the detection of Stx receptors in GSL preparations of LLC-PK1 and PK-15 cells and for binding studies with the neoglycolipids Gb3-PE and Gb4-PE, following a previously published protocol [77]. Murine anti-Stx1 and anti-Stx2 monoclonal antibodies of the IgG type (clone VT109/4-E9 and clone VT135/6-B9, respectively) were purchased from SIFIN GmbH (Berlin, Germany). Secondary goat anti-mouse IgG (no. 115-055-003) and rabbit anti-chicken IgY (no. 303-055-003), both labelled with alkaline phosphatase (AP), were from Dianova (Hamburg, Germany).

### 2.7. Thin-Layer Chromatography and Overlay Assay

A mixture of neutral GSLs from human erythrocytes containing the Stx receptor GSLs Gb3Cer and Gb4Cer, as well as minor amounts of lactosylceramide (Lc2Cer, Galβ1-4Glcβ1-1Cer), served as reference R1 [78]. A compilation of virtually equal amounts of Gb3Cer and Gb4Cer, prepared from human erythrocytes, is referred to as reference R2 [79]. Reference R2 has been generated by reducing the very high content of Gb4Cer using silica gel column fractionation by pooling fractions with high Gb3Cer and low Gb4Cer content. R2 harbors a nearly 1:1 molar ratio of Gb3Cer:Gb4Cer having the advantage that one can easily estimate a preference of a given Stx variant for Gb3Cer or Gb4Cer in thin-layer chromatography (TLC) overlay assays. The neutral GSL preparations from LLC-PK1 and PK-15 cells, reference GSLs and the neoglycolipids, Gb3-PE and Gb4-PE, were dispersed as 5 mm streaks onto glass-backed high-performance TLC plates precoated with silica gel 60 (Art. 1.05633.0001, Merck) with the aid of a Linomat 5 automatic sample applicator (CAMAG, Muttenz, Switzerland). Neutral GSLs and neoglycolipids were chromatographed in chloroform/methanol/water (120/70/17, *v/v*/v) and stained with orcinol. Immunochemical overlay detection of the Gb3 and Gb4 glycans with polyclonal chicken anti-Gb3Cer and anti-Gb4Cer antibodies, respectively, and detection of Stx-binding GSLs using Stx-containing supernatants from STEC liquid cultures combined with monoclonal anti-Stx antibodies (see Section 2.6. Stx1a, Stx2a, Stx2e and anti-Stx, anti-GSL, and secondary antibodies) were performed as described in a previous publication [80]. The monoclonal antibodies against Stx1 and Stx2 were diluted 1:1000, and the polyclonal antibodies against Gb3Cer and Gb4Cer, as well as the AP-conjugated secondary antibodies, were diluted 1:2000 for application in the TLC overlay assays. A solution of 0.05% (w/v) 5-bromo-4-chloro-3-indolyl phosphate (BCIP) was used as AP substrate for the detection of bound antibodies.

### 2.8. Structural Characterization of GSLs and Neoglycolipids by Mass Spectrometry

The structures of neutral GSLs of LLC-PK1and PK-15 cells (see Section 2.5. Isolation of neutral GSLs from LLC-PK1 and PK-15 cells) and produced neoglycolipids Gb3-PE and Gb4-PE (see Section 2.2. Preparation of the neoglycolipids Gb3-PE and Gb4-PE) were elucidated by nano-electrospray ionization mass spectrometry (nanoESI MS). To this end, GSLs and neoglycolipids were resolved in methanol and chloroform/methanol (2/8, *v/v*), respectively. The MS analysis was performed on a SYNAPT G2-S mass spectrometer (Waters, Manchester, UK) equipped with a Z-spray source in the positive ion sensitivity mode at a source temperature of 80 °C, a capillary voltage of 0.8 kV, a sampling cone voltage of 20 V, and a source offset voltage of 50 V. For low energy collision-induced dissociation (CID) measurements, quadrupole-selected precursor ions were fragmented in the trap cell using a collision gas (Ar) at a flow rate of 2.0 mL/min and collision energies up to 50 eV (E_lab_). For the MS analysis of overlay-detected Gal_2_Cer, the Plexigum (polyisobutylmethacrylate) silica gel fixative was removed with chloroform and the silica gel at the position of Stx-positive dihexosylceramide was scratched off the glass plate followed by GSL extraction as previously described [81]. Structures of individual GSLs were deduced from CID spectra, and the nomenclature of carbohydrate fragmentation according to Domon and Costello was utilized for the assignment of the fragment ions obtained by CID analysis [82,83].

## 3. Results

The first approach of this study was the demonstration of the occurrence of Gb3Cer and Gb4Cer, the eponymous GSLs of the globo-series, in neutral GSL fractions prepared from lipid extracts of cultured porcine LLC-PK1 and PK-15 kidney epithelial cell lines. Both GSLs are the canonical receptor GSLs of all Stx subtypes analyzed so far and their presence and structures have never been previously characterized in detail for LLC-PK1 and PK-15 cells. Two biological replicates of each cell line were employed for the following immunochemical analysis of the oligosaccharide portions of the Stx-binding GSLs and their structural characterization by ESI MS.

### 3.1. Immunochemical Detection of Gb3Cer and Gb4Cer in GSL Preparations of LLC-PK1 and PK-15 Cells

**Figure 1:**
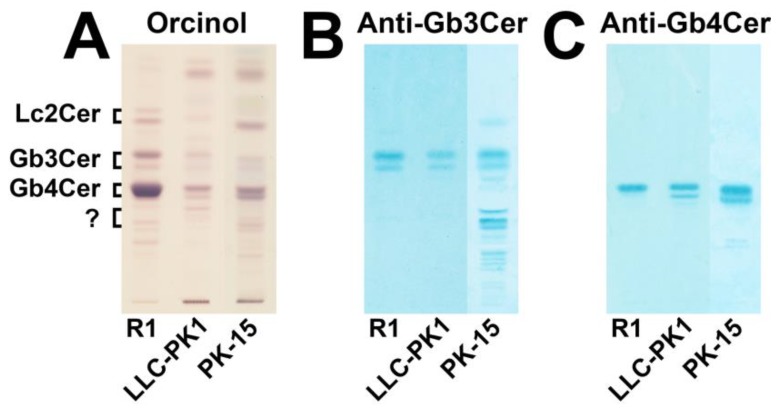
Orcinol stain (**A**) and antibody-mediated immunochemical detection of thin-layer chromatography (TLC) separated globo-series Shiga toxin (Stx) receptor glycosphingolipids (GSLs) Gb3Cer (**B**) and Gb4Cer (**C**) in the neutral GSL preparations of the porcine LLC-PK1 and PK-15 renal epithelial cell lines. The applied GSL quantities correspond to 2 × 10^6^ LLC-PK1 and 1 × 10^6^ PK-15 cells for the orcinol stain (**A**) and to 5 × 10^5^ LLC-PK1 and PK-15 cells for the anti-Gb3Cer (**B**) and anti-Gb4Cer overlay assay (**C**). R1: 20 µg (**A**), 2 µg (**B**), and 0.2 µg (**C**) of neutral GSLs from human erythrocytes served as reference.

Lipid extracts were prepared from the in vitro cultivated epithelial cells, and the neutral GSLs were isolated by means of anion-exchange chromatography. The orcinol stain of the TLC-separated GSLs and the overlay assays using the anti-Gb3Cer and anti-Gb4Cer antibody are shown in Figure 1. The orcinol stain revealed putative existence of small quantities of Gb3Cer and moderate amounts of Gb4Cer in both cell lines, which localized at the positions of Gb3Cer and Gb4Cer, respectively, of the reference GSL mixture R1 from human erythrocytes (Figure 1A). The anti-Gb3Cer overlay assay gave positive results and verified the presence of Gb3Cer in both cell lines (Figure 1B). In addition, a cross-reacting GSL, at the position of a suspected pentahexosylceramide with unknown structure separating just beneath Gb4Cer, and traces of a second unknown GSL, most likely a hexahexosylceramide, were detected in PK-15 cells. The anti-Gb4Cer antibody evidenced the existence of Gb4Cer in the two cell lines, suggesting higher content in PK-15 as compared with LLC-PK1 cells (Figure 1C). The same set of experiments was performed with a GSL preparation of the independent second biological replicate and gave identical results, which are displayed in Supplementary Figure S2. Thus, LLC-PK1 and PK-15 cells do express the classical globo-series GSLs, Gb3Cer and Gb4Cer, which migrate as double bands on the TLC plate suggesting some heterogeneity in the lipid ceramide portion of the respective GSL.

### 3.2. Identification of Stx-Binding GSLs Expressed by LLC-PK1 and PK-15 Cells

Similarity searches and comparison with *stx* reference sequences of the Stx subtypes of strains 2074/97, 03-06016, 2771/97, and S115G, corresponding to Stx1a, Stx2a, Stx2e(h), and Stx2e(p), respectively, used in this study was performed. Minor nucleotide sequence exchanges were detected as compared with the *stx* reference sequences [33] and are listed together with the corresponding amino acid exchanges in Table 1. In short, strains 2074/97 (Stx1a) and 03-06016 (Stx2a) each differ only in one non-synonymous single nucleotide polymorphism (SNP) in subunit A. Strain 2771/97 [Stx2e(h)] differs in two, non-synomymous SNPs, and strain S115G [Stx2e(p)] shows three SNPs as compared with reference sequences [33], all located in subunit A. No dissimilarities were detected for the respective B subunits.

**Figure 2:**
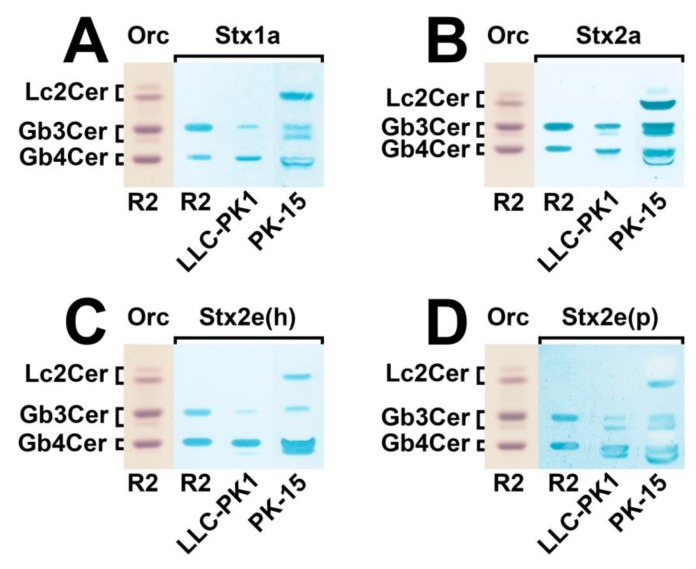
Detection of Stx-binding GSLs in the neutral GSL preparations of porcine LLC-PK1 and PK-15 renal epithelial cell lines. (**A**–**D**) Stx1a and Stx2a subtypes originated from human EHEC isolates. The two Stx2e variants are of different origin, Stx2e(h) derived from a human (h) and Stx2e(p) from a porcine (p) Stx-producing *Escherichia coli* (STEC) isolate. Applied GSL amounts correspond to 2 × 10^6^ cells for the Stx overlay assays. R2: 20 µg and 2.4 µg of an equimolar mixture of Gb3Cer and Gb4Cer served as reference for the orcinol (Orc) stains and the Stx overlay assays, respectively.

The TLC overlay assays of these Stx subtypes with GSLs of the two cell lines indicate a number of Stx-binding GSLs, as displayed in Figure 2. When compared to the reference R2, containing equimolar concentrations of Gb3Cer and Gb4Cer, the utilized Stx1a variant exhibited a slight preference for Gb3Cer as compared with Gb4Cer (Figure 2A). However, both GSLs were recognized by Stx1a exhibiting a faint positive Gb3Cer and a clear positive Gb4Cer band in case of LLC-PK1 cells. The PK-15 cells showed a weak Gb3Cer-positive doublet, a reasonable positive Gb4Cer binding, and a strong positive reaction with a GSL that co-separates at the position of reference lactosylceramide (Lc2Cer). Lc2Cer can be definitively excluded as attachment structure, suggesting the presence of a dihexosylceramide with Galα1-4Gal-oligosaccharide moiety (see below Section 3.4. Structural characterization of Stx receptor galabiosylceramide of PK-15 cells). The binding profile of the applied Stx2a variant was similar to Stx1a though indicating substantially more pronounced overlay color intensity (Figure 2B). The TLC overlay assay of the Stx2e(h) variant derived from an STEC strain of a human (h) patient is shown in Figure 2C. Stx2e is rather unusual as a source of Stx2e-producing STEC isolated from human stool, and therefore emphasized as “Stx2e(h)” in this context as compared to Stx1a- and Stx2a-producing EHEC strains, which are generally of human origin. Stx2e(h) revealed a preferred attachment to Gb4Cer as compared with Gb3Cer, as shown by the interaction with reference R2 composed of identical G3Cer and Gb4Cer molarities (Figure 2C). Its preference for Gb4Cer can be also deduced from binding towards LLC-PK1- and PK-15-derived GSLs due to the most intensive Gb4Cer bands in both cell lines. Owing to the generally favored binding of Stx2e to elongated globo-series GSLs, the interaction toward the Stx-binding disaccharide was considerably inferior as compared with Stx1a and Stx2a (cf. Figure 2A,B, respectively). Stx2e(p) obtained from an STEC strain of porcine (p) origin exhibited the same Gb4Cer prevalence as Stx2e(h), but showed a clear double band pattern for Gb3Cer of LLC-PK1 and PK-15 and for Gb4Cer of LLC-PK1 cells (Figure 2D) in contrast to Stx2e(h) (Figure 2C). Adhesion assays of the employed Stx subtypes performed with GSL preparations derived from the second biological replicate of LLC-PK1 and PK-15 cells corroborated their distinct binding specificities, as shown in the Supplementary Figure S3. Importantly, none of the investigated Stx subtypes recognized any of the anti-Gb3Cer-crossreactive GSLs (cf. Figure 1B), indicating their irrelevance regarding the Stx recognition process. Thus, the presence of Gb5Cer or Forssman GSL, known as specific and favored receptor GSLs of Stx2e subtype [30,32], in the GSL preparations of LLC-PK1 and PK-15 cells, can be excluded. However, the proposed Stx-binding GSLs require a precise mass spectrometric proof, which is given in the next paragraph.

### 3.3. Structural Characterization of Stx Receptors Gb3Cer and Gb4Cer of LLC-PK1 and PK-15 Cells

The exact structures of hypothesized Stx receptor GSLs, Gb3Cer and Gb4Cer, of the porcine LLC-PK1 and PK-15 cell lines were scrutinized by means of ESI MS using the positive ion mode. Due to the superior resolution, the applied technique is ideal to determine the various lipoforms and the oligosaccharide sugar sequence of immunochemically identified glycan epitopes of Gb3Cer and Gb4Cer (cf. Figure 1B,C). More precisely, MS^1^ and MS^2^ analysis of GSLs allows for unravelling the monosaccharide composition (hexoses, *N*-acetylhexosamines or desoxyhexoses) and sequence of the sugars, as well as the lipid composition of the two-tailed ceramide moiety of a GSL. In mammalian cells, the ceramide portion usually consists of an invariable dihydroxylated monounsaturated C18 long-chain base, called sphingosine (d18:1), connected with an amide-bound fatty acid that can vary in chain length (C16 to C24) and degree of unsaturation (e.g., C24:0 versus C24:1). The MS^1^ analysis provides an overview on the composition of a GSL mixture. The MS^2^ analysis comprises in-depth fragmentation analysis of a selected GSL using, for instance, a technique termed collision-induced dissociation (CID). However, completion of the structural analysis of a GSL requires the determination of the linkage positions (e.g., 1-3, 1-4, or others) and the anomeric configuration (α versus β configuration) of the individual monosaccharides in the glycan chain. This can be accomplished by mono- or polyclonal antibodies each exhibiting a certain glycan binding specificity towards, for example, the Gb3 or Gb4 oligosaccharide. For determining the specific sugars forming the characteristic oligosaccharide configuration, for example, of the Gb3 oligosaccharide with Galα1-4Galβ1-4Glc structure, an antibody is required that specifically recognizes this epitope. The reason for the immunodetection is the fact that the MS analysis alone provides precise information about the Hex-Hex-Hex-Cer structure including the exact molecular weight, the monosaccharide sequence, and the composition of the ceramide moiety, but no further structural details on the glycan portion. Thus, MS is unable to determine the hexose type (e.g., galactose versus glucose) and linkage positions of the monosaccharides (1-3 versus 1-4) and cannot discriminate between α- and β-configuration. Therefore, we usually combine TLC separation of GSLs with antibody-mediated overlay detection and deepened structural investigation using ESI MS^1^ and MS^2^ analysis [84,85], as demonstrated in this publication for structural investigations on Stx-binding GSLs of porcine epithelial cells (see below). Thus, the MS^1^ analysis revealed mass spectra of the Gb3Cer and Gb4Cer species of the porcine epithelial cell lines, whereas the MS^2^ analysis of the individual GSLs allowed for monosaccharide sequencing of the oligosaccharide portions and determining the ceramide structure regarding sphingosine and fatty acid composition, and anti-Gb3Cer and anti-Gb4Cer antibodies identified the Gb3 and Gb4 oligosaccharides, respectively.

#### 3.3.1. Gb3Cer and Gb4Cer Lipoforms of LLC-PK1 Cells

**Figure 3:**
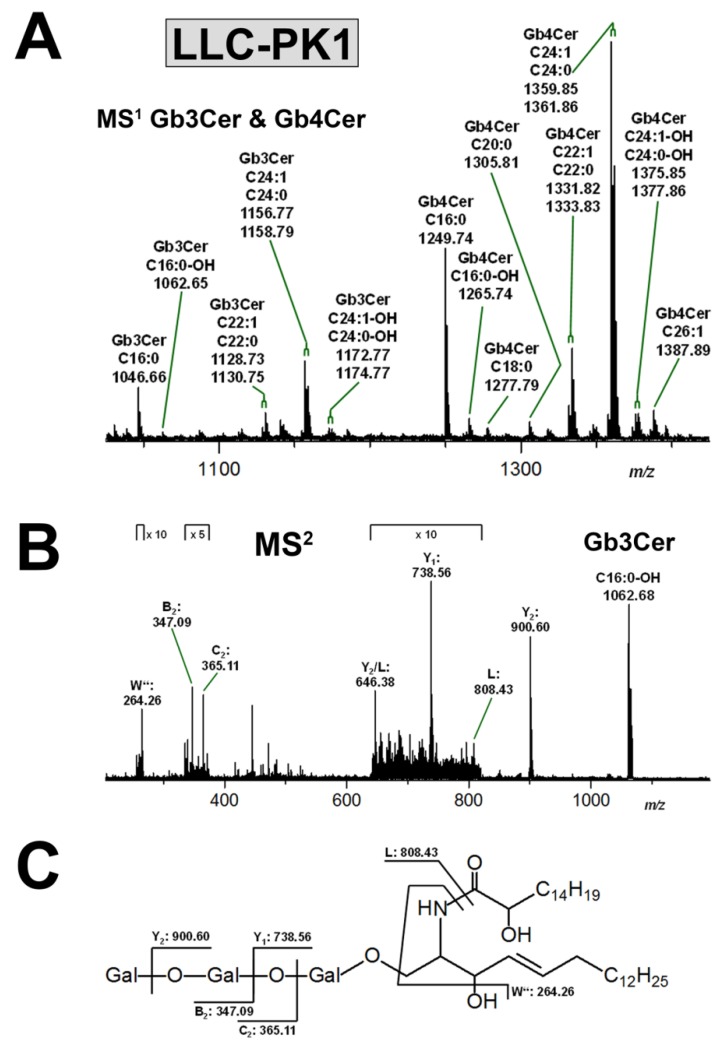
Overview mass spectrometry (MS)^1^ spectrum of the Stx receptor GSLs Gb3Cer and Gb4Cer (**A**) and MS^2^ spectrum of selected Gb3Cer (d18:1, C16:0-OH) species (**B**) with the auxiliary fragmentation scheme (**C**) obtained from porcine LLC-PK1 kidney epithelial cells. The MS^1^ spectrum spans the *m/z* range between 1030 and 1430 showing Gb3Cer and Gb4Cer lipoforms with ceramide moieties composed of an invariable sphingosine (d18:1) and variable fatty acids, which ranged in chain length from C16 to C26 as assigned in the spectrum. All GSLs were detected as monosodiated species ([M+Na]^+^) using the positive ion mode, and are listed in Supplementary Table S1. An MS^2^ structural proof of MS^1^-based proposed structures is exemplarily provided for the very low abundant hydroxylated Gb3Cer (d18:1, C16:0-OH) species, demonstrating the exceptional performance of the employed MS technology.

The mass spectrum covering the *m/z* range of the proposed Gb3Cer and Gb4Cer species is shown in Figure 3A and the identified GSLs are listed in Supplementary Table S1. All GSL species are detected in their sodiated form, i.e., as [M+Na]^+^ ions. Apparent divergent lipoforms derive from different ceramides composed of d18:1 sphingosine (more precisely and systematically denoted as “sphingenine”) as the invariable portion and fatty acids with acyl chains ranging from C16 to C26 as variable moieties as indicated in the spectrum. Gb3Cer and Gb4Cer with Cer (d18:1, C16:0), Cer (d18:1, C22:0), and Cer (d18:1, C24:1/C24:0) can be considered the major Stx receptor GSLs of LLC-PK1 cells, whereas Gb4Cer species, noticeably dominate over those of Gb3Cer, can be deduced from the differing ion abundancies. As an example for a firm and detailed structural analysis of a very minor GSL species, Figure 3B portrays the MS^2^ spectrum of Gb3Cer (d18:1, C16:0-OH) together with an explanatory fragmentation scheme in Figure 3C. Fragment ions formed by cleavages within the oligosaccharide part were annotated according to the nomenclature of Domon and Costello [82,83], whereas ionic species arising from fragmentation of the ceramide were assigned following the nomenclature by Hsu et al. [86]. Evaluation of the MS^2^ spectrum reveals the presence of a hydroxylated fatty acid and, by the way, demonstrates the outstanding achievement potential of sophisticated state-of-the-art MS analysis.

#### 3.3.2. Gb3Cer and Gb4Cer Lipoforms of PK-15 Cells

**Figure 4:**
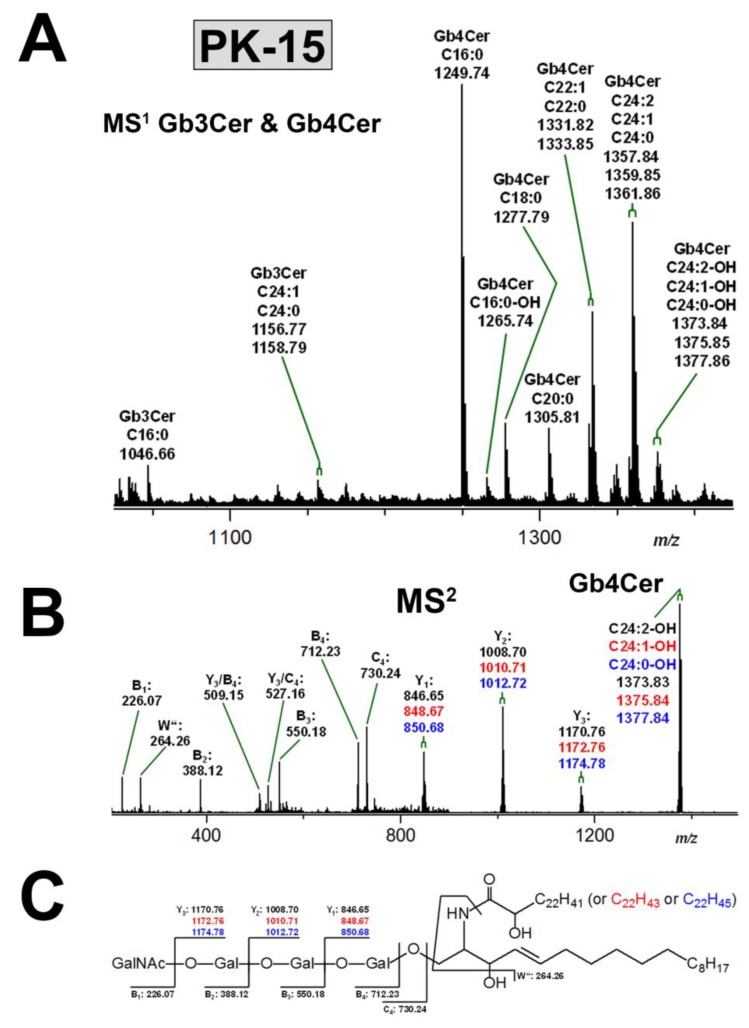
Overview MS^1^ spectrum of the Stx receptor GSLs Gb3Cer and Gb4Cer (**A**) and MS^2^ spectrum of the selected Gb4Cer triplet composed of Gb4Cer lipoforms harboring Cer (d18:1, C24:2-OH), Cer (d18:1, C24:1-OH), and Cer (d18:1, C24:0-OH), respectively, (**B**) with the auxiliary fragmentation scheme (**C**) obtained from porcine PK-15 kidney epithelial cells. The MS^1^ spectrum spans the *m/z* range between 1030 and 1430 showing Gb3Cer and Gb4Cer lipoforms with ceramide moieties composed of an invariable sphingosine (d18:1) and variable fatty acids, which ranged in chain length from C16 to C24 as assigned in the spectrum. All GSLs were detected as monosodiated species ([M+Na]^+^) using the positive ion mode and are listed in Supplementary Table S2. An MS^2^ structural proof of MS^1^-based proposed structures is exemplarily provided for the highly variable hydroxylated Gb4Cer species carrying two-fold and one-fold unsaturated and saturated C24:2, C24:1, and C24:0 fatty acid, respectively, in the ceramide lipid anchor showing the exceptional discriminatory power of state-of-the-art MS analysis.

Figure 4A depicts the mass spectrum of the proposed Gb3Cer and Gb4Cer species obtained from PK-15 cells detected in the relevant *m/z* range. Again, all GSL species appear as [M+Na]^+^ ions and their identified structures are listed in Supplementary Table S2. The observed heterogeneity of the GSL species is due to ceramides with constant d18:1 sphingosine but varying fatty acid chain length, as described in the preceding chapter for the Gb3Cer and Gb4Cer lipoforms of LLC-PK1 cells. Owing to the apparent signal intensities, ceramides with (d18:1, C16:0), (d18:1, C22:0), and (d18:1, C24:1/C24:0) structure are the prevalent lipid anchors of the various Gb3Cer and Gb4Cer species. Gb4Cer-derived ions strictly preside over less abundant Gb3Cer species in the MS^1^ spectrum and may represent the major Stx receptor GSLs of PK-15 cells. An exemplary MS^2^ spectrum demonstrating the high resolution capacity of the employed MS strategy is given in Figure 4B, unravelling the structures of the individual variants of the Gb4Cer triplet consisting of hydroxylated di- and mono-unsaturated and saturated variants with Cer (d18:1, C24:2-OH), Cer (d18:1, C24:1-OH), and Cer (d18:1, C24:0-OH) lipoforms, respectively. The CID spectrum is explained in the accompanying fragmentation scheme in Figure 4C.

### 3.4. Structural Characterization of Stx Receptor Galabiosylceramide of PK-15 Cells

**Figure 5:**
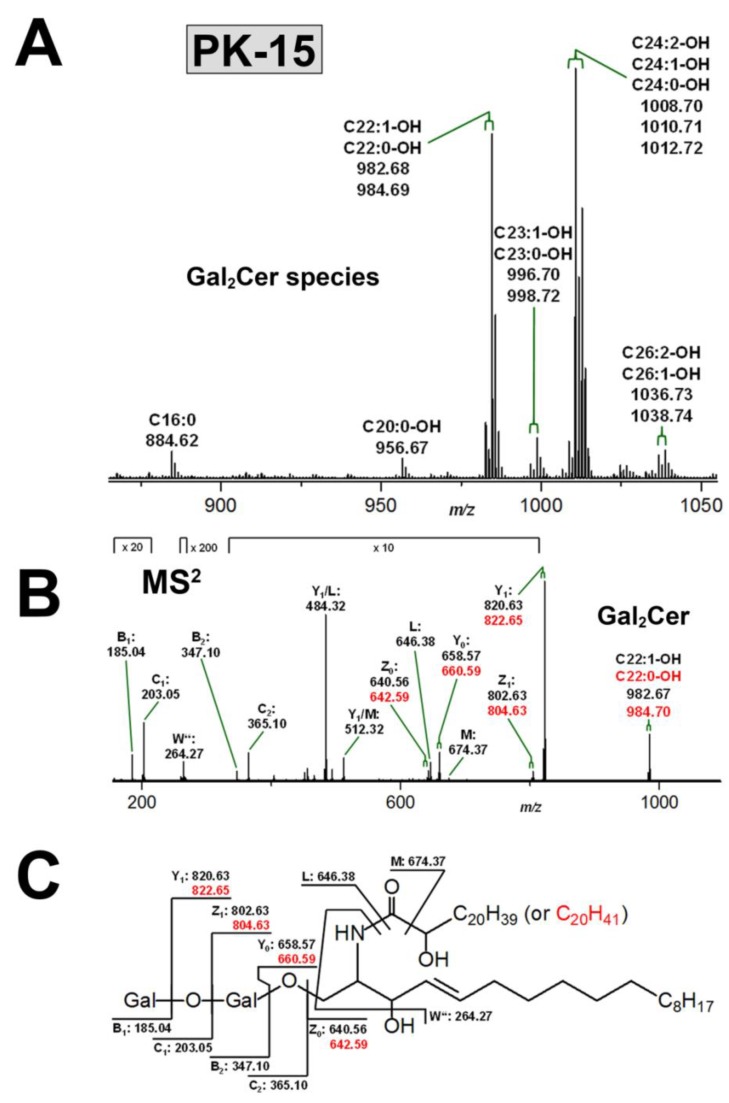
Overview MS^1^ spectrum of Stx receptor Gal_2_Cer species (**A**) and MS^2^ spectrum of selected Gal_2_Cer (d18:1, C22:1-OH)/Gal_2_Cer (d18:1, C22:0-OH) doublet (**B**) with the auxiliary fragmentation scheme (**C**) obtained from porcine PK-15 kidney epithelial cells. The MS^1^ spectrum spans the *m/z* range between 865 and 1055 showing Gal_2_Cer lipoforms with ceramide moieties composed of a consistent sphingosine (d18:1) and variable fatty acids, which ranged in chain length from C16 to C26 as assigned in the spectrum. All GSLs were detected as monosodiated species ([M+Na]^+^) ions using the positive ion mode and are listed in Supplementary Table S3. An MS^2^ structural proof of MS^1^-based proposed structures is exemplarily provided for the Gal_2_Cer species with hydroxylated one-fold unsaturated and saturated C22:1 and C22:0 fatty acid, respectively, showing again the outstanding discriminatory power of the employed state-of-the-art MS technology.

The Stx-recognized GSLs of PK-15 cells that separate at the TLC position of Lc2Cer (see Figure 2) and putatively identified as dihexosylceramides with Galα1-4Gal-sequence (see above 3.2. Identification of Stx-binding GSLs expressed by LLC-PK1 and PK-15 cells) underwent concise MS analysis. To this end, the silica gel of the Stx2a-positive bands of the two biological replicates of PK-15 cells (see Figure 2B and Supplementary Figure S3B) was scraped, the GSLs extracted from the gel and subjected to MS analysis, as shown in Figure 5. The obtained MS^1^ spectrum revealed [M+Na]^+^ ions corresponding to hydroxylated dihexosylceramides mostly with Cer (d18:1, C22:1-OH) and Cer (d18:1, C22:0-OH), as well as Cer (d18:1, C24:2-OH), Cer (d18:1, C24:1-OH), and Cer (d18:1, C24:2-OH), as shown in Figure 5 and listed in Supplementary Table S3. These prevalent structures were accompanied by minor proposed Gal_2_Cer lipoforms carrying Cer (d18:1, C23:1-OH) and Cer (d18:1, C23:0-OH), as well as Cer (d18:1, C26:2-OH), and Cer (d18:1, C26:1-OH) lipid anchors. Gal_2_Cer (d18:1, C16:0) was the only non-hydroxylated species. Thus, the detected heterogeneity of the Gal_2_Cer species is due to uniform d18:1 sphingosine combined with fatty acids of chain lengths, as described in the preceding chapter for the non-hydroxylated Gb3Cer and Gb4Cer lipoforms of PK15 cells (see 3.3.2 Gb3Cer and Gb4Cer lipoforms of PK-15 cells). The MS^2^ analysis corroborated the proposed structures and an exemplary MS^2^ spectrum of the Gal_2_Cer doublet with hydroxylated Cer (d18:1, C22:1-OH) and Cer (d18:1, C22:0-OH) moieties supported by the explanatory fragmentation scheme are provided in Figure 5B,C, respectively.

### 3.5. Neoglycolipids as Receptors and Potential Inhibitors of Stxs

**Figure 6:**
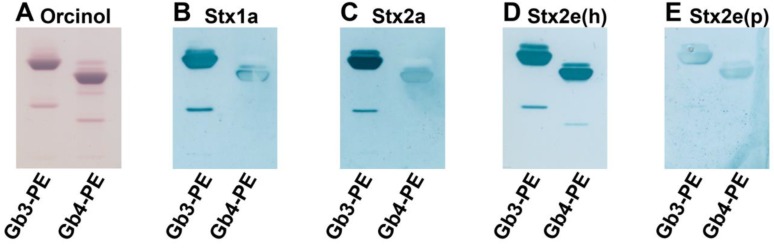
Orcinol stain (**A**) and Stx overlay detection (**B**–**E**) of TLC-separated neoglycolipids Gb3-PE and Gb4-PE. Stx1a and Stx2a subtypes originated from human EHEC isolates. The two variants of Stx2e subtype derived from human (h) and porcine (p) STEC isolates were differentiated in Stx2e(h) and Stx2e(p), respectively. Amounts of Gb3-PE and Gb4-PE were 5 µg, respectively, for the orcinol stain (**A**) and each Stx overlay assay (**B**–**E**).

Targeting the synthesis of defined and homogenous glycolipids with Gb3- or Gb4-headgroups as a diagnostic tool for differentiation of the binding specificities of the various Stx subtypes or for employment as neutralizers of Stx cytotoxicity, a semisynthetic approach was chosen for the production of Gb3- and Gb4-neoglycolipids harboring a stable and consistent lipid anchor. The orcinol stain of Gb3-PE and Gb4-PE, produced from commercially available Gb3 and Gb4 oligosaccharides by coupling them to twin-tailed phosphatidylethanolamine (PE), is shown in Figure 6A. The minor sharp lower migrating bands are derived from neoglycolipids containing two oligosaccharide moieties, i.e., (Gb3)_2_-PE and (Gb4)_2_-PE, respectively. Corresponding compounds are commonly obtained as byproducts of the coupling reaction in the lower single-digit percentage range. Stx1a and Stx2a bound preferably to Gb3-PE and with less affinity towards Gb4-PE, as can be deduced from the TLC overlay assays depicted in Figure 6B,C, respectively. A shift of preference towards interaction with Gb4-PE was observed for Stx2e(h) and Stx2e(p), as shown in Figure 6D,E, respectively. Interestingly, the Stx2e(p) variant exhibited vastly less recognition intensity as compared with the human Stx2e(h) counterpart.

### 3.6. Structural Characterization of Stx-Binding Neoglycolipids Gb3-PE and Gb4-PE

**Figure 7:**
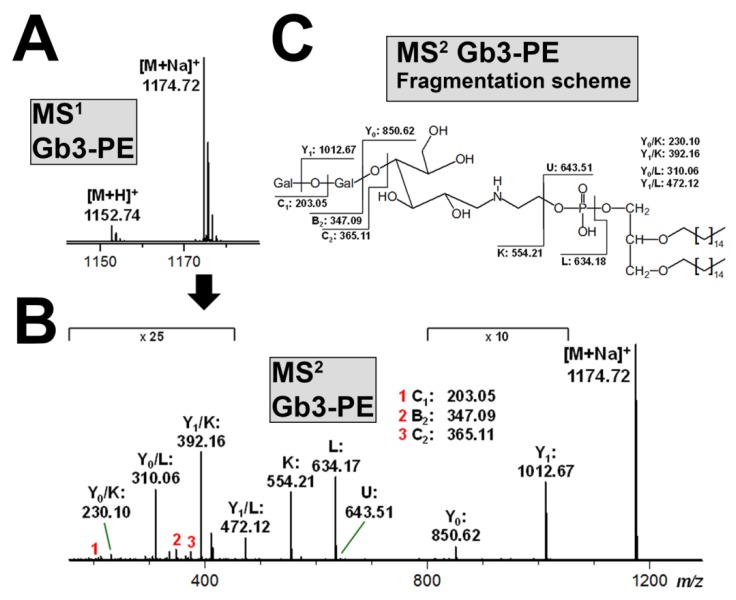
MS^1^ spectrum (**A**) and MS^2^ structural proof (**B**) with corresponding fragmentation scheme (**C**) of the produced neoglycolipid Gb3-PE. MS analysis using the positive ion mode exhibits dominance of monosodiated [M+Na]^+^ ions over the protonated [M+H]^+^ ion species (**A**). The [M+Na]^+^ ions at *m/z* 1174.72 were selected for MS^2^ analysis (**B)** and the obtained breakdown products are explained by the auxiliary fragmentation scheme (**C**).

The purity of the produced neoglycolipids was examined by means of ESI MS^1^ and MS^2^ analysis working in the positive ion mode. The MS^1^ spectrum of synthesized Gb3-PE revealed [M+Na]^+^ ions at *m/z* 1174.72 as the most prominent analyte species as compared with the protonated counterparts ([M+H]^+^) appearing as less abundant ion species at *m/z* 1152.74, as demonstrated in Figure 7A. The proposed structure of Gb3-PE, based on the glycerol core carrying two identical C16 alkyl chains and a phosphate group, which links the glucose molecule of the reducing end of the Gb3 oligosaccharide via the ethanolamine group, was confirmed by the MS^2^ analysis, as shown in Figure 7B, accompanied by the explanatory fragmentation scheme in Figure 7C.

**Figure 8:**
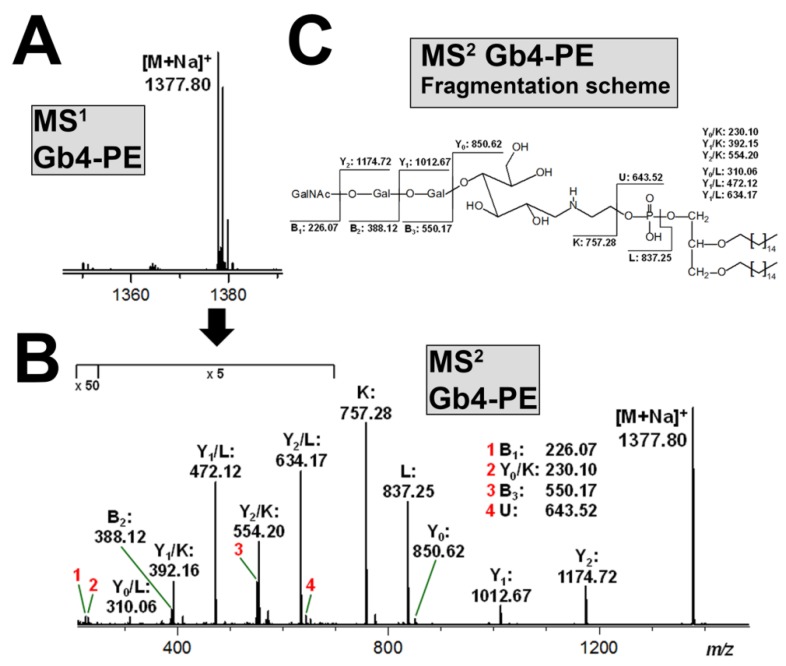
MS^1^ spectrum (**A**) and MS^2^ structural proof (**B**) with corresponding fragmentation scheme (**C**) of produced Gb4-PE. The MS analysis using the positive ion mode exhibits solely monosodiated species ([M+Na]^+^) (**A**). The [M+Na]^+^ ions at *m/z* 1377.80 served as precursor ions for the MS^2^ analysis (**B**) explained by the supporting fragmentation scheme (**C**).

The same PE lipid anchor was linked to Gb4, and the MS^1^ spectrum of the resulting neoglycolipid Gb4-PE is given in Figure 8A. The spectrum indicated the sole presence of monosodiated species ([M+Na]^+^) at *m/z* 1377.80. The CID experiments confirmed the proposed structure of C16 twin-tailed Gb4-PE, as shown in Figure 8B, and the fragmentation scheme in Figure 8C explains the complete structure derived from the fragment ions.

### 3.7. Protection of Kidney Epithelial Cells from Stx2e-Mediated Damage by Gb3-PE and Gb4-PE

**Figure 9:**
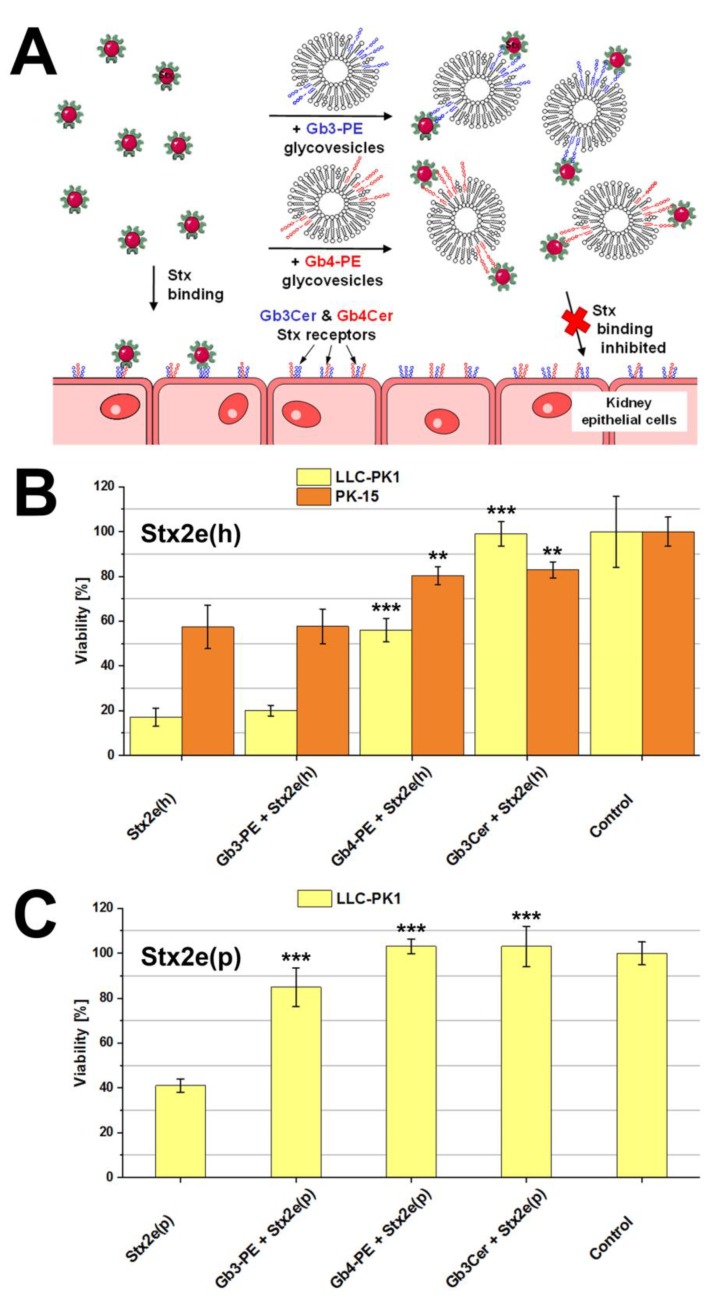
Scheme of attachment of Stx2e to the cellular receptors Gb3Cer and Gb4Cer, its interaction with Gb3-PE and Gb4-PE in the cell culture (**A**) and protective effect of the neoglycolipids towards porcine LLC-PK1 and PK-15 kidney epithelial cells exerted by Stx2e(h) (**B**) and Stx2e(p) (**C**). (**A**) Gb3-PE- and Gb4-PE-spiked glycovesicles were applied to block the binding of Stx2e towards Gb3Cer and Gb4Cer. (**B**) Stx2e(h) derived from a human STEC isolate and (**C**) Stx2e(p) from a porcine STEC isolate were solely applied or together with the neoglycolipids Gb3-PE and Gb4-PE or with authentic Gb3Cer (gold standard) as indicated. Cell survival was determined in relation to control cells cultured in medium without toxin corresponding to 100% viability. Significant differences of viability of cell cultures treated with Stx2e only (controls) or co-treated with Gb3-PE-, Gb4-PE- or Gb3Cer-spiked glycovesicles are indicated with asterisks (** *p* < 0.01; *** *p* < 0.001).

The scheme provided in Figure 9A portrays the principal interaction of Stx with cellular Gb3Cer and Gb4Cer exposed on the surface of kidney epithelial cells and the interplay of Stx with applied glycovesicles, suggesting binding and inhibition of the toxin by Gb3-PE- and/or Gb4-PE-spiked glycovesicles. Both LLC-PK1 and PK-15 cells were sensitive towards Stx2e(h) when treated with the toxin alone, as shown in Figure 9B. Exposure to Stx2e(h) resulted in significantly decreased viability of 16.5% ± 4.0% in case of LLC-PK1 and a cell viability drop down to 57.4% ± 9.7% of PK-15 cells in comparison to 100.0% ± 9.1% and 100.0% ± 3.7% viability of untreated cells (controls), respectively. Application of glycovesicles spiked with authentic Stx receptor Gb3Cer (gold standard) together with the toxin inhibited Stx2e(h)-mediated cell killing resulting in 99.0% ± 5.5% viability of LLC-PK1 and 82.9% ± 4.1% viability of PK-15 cells (Figure 9B). The exposure of Stx2e(h) and the neoglycolipid Gb4-PE bound to glycovesicles resulted in more than tripling the viability of LLC-PK1 cells to 56.5% ± 5.3% and an increased viability of factor 1.4 in the case of PK-15 cells reaching an 80.4% ± 3.6% viability as compared with the cells exposed to Stx2e(h) alone, whereas Gb3-PE exhibited no protection against the toxin for both cell types (Figure 9B).

Treatment of LLC-PK1 cells with porcine-derived Stx2e(p) led to reduced cell viability of 41.2% ± 3.0% (Figure 9C), whereas PK-15 resisted towards Stx2e(p) (not shown). Thus, neutralization studies with glycovesicles were performed with LLC-PK1 cells only. The incubation of LLC-PK1 cells with Stx2e(p) and glycovesicles loaded with genuine Stx receptor Gb3Cer (gold standard) gave complete neutralization of Stx2e(h) resulting in 102.6% ± 9.0% survival compared to 100.0% ± 5.1% viability of untreated cells (controls). A virtually identical rate of detoxification was achieved with Gb4-PE-spiked glycovesicles amounting to 103.1% ± 3.4% viability (Figure 9C). Furthermore, exposure of LLC-PK1 cells with Stx2e(p) together with glycovesicles containing Gb3-PE eventuated in 84.9% ± 8.6% viability corresponding to more than duplication of cell survival in comparison to cells exposed to Stx2e(p) only (Figure 9C).

Collectively, on the one hand, the produced neoglycolipid Gb4-PE significantly reduced the cytotoxic activity of Stx2e(h) towards PK-15 and LLC-PK1 cells, whereas Gb3-PE did not exhibit any positive effect. On the other hand, both Gb3-PE and Gb4-PE substantially reduced the cell killing activity of Stx2e(p) towards LLC-PK1 cells. Interestingly, PK-15 cells were refractory to Stx2e(p), wherefore inhibition experiments with the neoglycolipids became superfluous for this cell type.

## 4. Discussion

Starting with the first reports on preliminary data of the content of globo-series GSLs in porcine organs including kidney [23,24,25], the precise structures of Stx2e-binding and largely abundant Gb3Cer and Gb4Cer species were later on scrutinized in a number of tissues and organs including kidney cortex, medulla, and pelvis of weaning piglets [31]. The porcine kidney epithelial cell lines, LLC-PK1 and PK-15, analyzed in this study are not identical with the original tissue in that the cell lines exhibited a higher content of Gb4Cer versus Gb3Cer. Of note, a significant content of hydroxylated lipoforms of both, Gb3Cer and Gb4Cer, has been previously detected in the kidney, whereas this ceramide modification of the globo-series GSLs was not found in renal LLC-PK1 and PK-15 cells. However, hydroxylated Gb3Cer and Gb4Cer lipoforms have been hitherto found in porcine brain endothelial cells [7], whereas the biological relevance remains unknown. Notably, porcine kidney cortex, medulla, and pelvis were devoid of the Forssman GSL [31], which was also undetectable in LLC-PK1 and PK-15 cells. This is remarkable, because the Forssman GSL is a specific GSL receptor of swine-pathogenic Stx2e, but not of human-pathogenic Stx1a and Stx2a [30]. Regarding the general distribution in swine tissues and organs, very minute quantities of the Forssman GSL have been previously detected in muscle and jejunum only, suggesting its lack of relevance for Stx2e-mediated kidney injury. So far, the two Stx2e-binding pentahexosylceramides, namely the Forssman GSL and Gb5Cer, representing elongated Gb4Cer structures but with distinct sugar epitopes at the non-reducing end of the glycan moiety, have been only detected in kidney cell lines of canine and monkey origin, respectively. The Forssman GSL is a characteristic membrane constituent of the canine kidney epithelial cell line MDCK II of cocker spaniel origin [77] and Gb5Cer was found in a monkey kidney-derived Vero epithelial cell line [32].

Despite a similarly high content of Gb3Cer and Gb4Cer being recognized by Stx2e in both LLC-PK1 and PK-15 cells, the two cell lines differed significantly regarding their sensitivity towards Stx2e. LLC-PK1 cells were highly sensitive to Stx2e(h) derived from a human- and to Stx2e(p) released by a porcine-pathogenic STEC strain, whereas PK-15 cells were less susceptible towards Stx2e(h) and de facto resistant towards Stx2e(p). Comparison of the two *stx2e* sequences of strains 2771/97 [Stx2e(h)] and S115G [Stx2e(p)] revealed in total, three SNPs at positions 33, 887, and 937, the latter two with non-synonymous base exchanges (see Table 1). At the current stage of research, it remains unclear whether those minor dissimilarities might account for the observed differences in Stx-mediated cellular damage caused by the two Stx2e variants. However, the distinct susceptibility of the two cell lines might rest upon different modes of binding, uptake or retrograde transport of the toxic cargo to the intracellular targets. Thus, first the question of segregation in “lipid rafts” has to be addressed in future investigations. Lipid rafts exist in the outer leaflet of the plasma membrane of mammalian cells featuring microdomains, which are enriched in GSLs, sphingomyelin, and cholesterol [87]. The accumulation of GSL receptors in lipid rafts seems to be a pivotal requirement for efficient attachment and intracellular uptake of Stxs [88,89,90]. Moreover, exposure of Stx-binding Gb3Cer in lipid rafts has been reported to be involved in pathological events that occur during the development of HUS [91] and it has been postulated that the occurrence of Gb3Cer in lipid rafts of glomerular cells in the human kidney may be the reason for the glomerular-restricted pathology of HUS caused by Stxs [88,92]. A precise analysis of the lipid environment of Stx-binding GSLs of LLC-PK1 and PK-15 cells is, therefore, pending using, for example, lipid raft-analog detergent-resistant membranes resembling the liquid-ordered membrane phase [93,94]. Detergent-resistant membranes can readily be isolated which have many properties expected of lipid rafts [95] and represent useful biophysical approaches in the study of biomembrane lateral inhomogeneity [96]. The employment of this procedure for characterization of the lipid composition of such lipid raft-resembling membrane microdomains prepared from Stx-sensitive endothelial cells has been recently reviewed [97]. Second, different modes of uptake and cellular entry of Stx into the endosomal system have been published [89,98]. Moreover, the increasing degree of complexity of endocytotic processes and various intracellular trafficking routes of the toxin to subcellular targets have to be taken into account [99,100]. Third, besides the renowned inhibitory effects on protein biosynthesis by depurination of a certain adenosine residue of the ribosomal RNA, executed by the *N*-glycosidase activity of the toxin’s A1 fragment [101], damage to nuclear DNA by removal of multiple adenines when acting on DNA has to be considered as well [102,103]. Furthermore, induction of host cellular stress response resulting in apoptotic cell death has been recognized [104]. Thus, a number of factors might be involved and contribute to different effects exerted by Stxs that may result in high or low susceptibility or even in resistance towards Stxs, especially in view that different cell types, for example, endothelial and epithelial cells, might respond differently to the various Stx subtypes. To sum up, we cannot absolutely exclude that also minute contaminants in the Stx2e preparations might be involved in the differences observed in the cytotoxicity assays. However, the inhibition of the cytotoxic action of Stx2e using Gb3Cer (gold standard) and the neoglycolipid Gb4-PE strongly supports the assumption of Stx as the major virulence factor and cytotoxic compound in the supernatants of the employed strains.

Similar to the swine LLC-PK1 and PK-15 renal epithelial cell lines, the canonical globo-series Gb3Cer and Gb4Cer have been previously identified by us in the human T24 bladder [105] and A498 kidney epithelial cells, as recently published [79]. The human cell lines exhibited divergent lipoforms harboring the mentioned two GSLs with Cer (d18:1, C24:1/C24:0), Cer (d18:1, C22:0), and Cer (d18:1, C16:0) as the prevalent molecular species according to ion signal intensities determined by ESI MS analysis. Noteworthy, Gb3Cer and Gb4Cer of the human A498 kidney epithelial cells, showing a similar repertoire of globo-series GSLs like LLC-PK1 and PK-15 cells, served as efficient receptor GSLs for P-fimbriated uropathogenic *E. coli* (UPEC), the primary cause of urinary tract infections in humans [106,107]. P-fimbriae are fitted with the Gb3Cer- and Gb4Cer-binding PapG adhesins I, II, or III [108,109]. These three PapG classes were found to adhere differently to host cell Gb3Cer and Gb4Cer of human A498 cells, whereas PapG I exhibited equal binding strength to Gb3Cer and Gb4Cer, but PapG II and PapG III preferred Gb4Cer over Gb3Cer, with PapG III as the strongest binding partner for Gb4Cer [79]. Thus, pending interaction studies using GSL preparations of porcine renal LLC-PK1 and PK-15 cells will improve our knowledge on the molecular mechanisms of P-fimbriae-mediated adhesion of swine-pathogenic UPEC strains towards the porcine uroepithelium.

The rational design of soluble multivalent Stx inhibitors is a very real prospect to target the Gb3Cer-Stx recognition event, and a number of promising Stx receptor analogues, comprising the Gb3 trisaccharide linked to various scaffolds, has been developed [110,111,112]. Such semi-synthetic toxin binders have been demonstrated in animal models of STEC disease to be effective [113]. With the goal in mind of producing defined and homogenous glycolipids with Gb3- or Gb4-headgroups as ligands for the various Stx-subtypes including Stx2e and for usage as neutralizers of Stx-mediated cellular damage, a semi-synthetic approach was chosen by us for the production of Gb3- and Gb4-neoglycolipids harboring a stable and consistent lipid anchor. This attempt is superior due to limited resources of native Gb3Cer or Gb4Cer preparations, which can be isolated from human or animal organs as starting material but need further extensive column purification steps. Batch-to-batch heterogeneity is an additional uncertainty among a number of drawbacks working with GSLs derived from natural mammalian resources. Probing of produced Gb3-PE and Gb4-PE neoglycolipids as recognition structures in TLC overlay assays revealed strong interaction of human-pathogenic Stx1a and Stx2a towards Gb3-PE, accompanied by weak binding to Gb4-PE. In contrast to this, Stx2e bound equally well to Gb3-PE and Gb4-PE, although its preference for Gb4Cer when compared to recognition of Gb3Cer has been reported [28,29,30]. When applied as multivalent recognition structures in glycovesicles in this study, the neoglycolipids Gb3-PE and Gb4-PE exhibited substantial but different capability for detoxification of Stx2e. Highly sensitive LLC-PK1 cells were significantly protected against Stx2e(h) by Gb4-PE and the gold standard Gb3Cer, but not by Gb3-PE. The same holds true for the less susceptible PK-15 cells. Of note, the PK-15 cells were resistant towards Stx2e(p) but sensitive towards Stx2e(h) and could be protected by Gb4-PE and the gold standard Gb3Cer against Stx2e(h). The reason for this diversity remains elusive and, as mentioned above, several yet unknown factors might be responsible for the observed discrepancy that cannot be explained by our current state of knowledge. As an additional approach aimed at neutralizing Stx2e-mediated cellular damage, we probed the recently produced pectin-derived (α1-4)Gal_n_-PE neoglycolipids with 2 ≤ n ≤ 6, where “n” stands for the number of α1-4-linked galactose molecules [72]. Stx1a and Stx2a, the Stx subtypes of human relevance, bound in a characteristic and different manner to the various (α1-4)Gal_n_-PEs. Stx1a preferred neoglycolipids with short oligosaccharide chains, whereas Stx2a exhibited preference for those with longer glycans. However, the various Stx2e variants analyzed in that study showed only marginal binding to the (α1-4)Gal_n_-PE type neoglycolipids [72]. While the mixture of produced (α1-4)Gal_n_-PEs, which were applied as neoglycolipid-spiked glycovesicles, considerably diminished the cytotoxic activity of Stx1a and Stx2a towards Vero cells, Stx2e was not affected as expected from weak binding detected in the TLC overlay assays. In this study, the employed Stx2e(h) and Stx2e(p) variants revealed poor binding towards the (α1-4)Gal_n_-PEs variants (as expected) and the approach utilizing them as Stx neutralizers for porcine LLC-PK1 and PK-15 kidney epithelial cells failed completely (not shown).

## 5. Concluding Remarks

Shiga toxins (Stxs) of subtype Stx2e derived from a certain pathogenic subgroup of *Escherichia coli* bacteria cause the edema disease in pigs. Stx2e gains access from the intestine to the circulation, where the toxin causes severe kidney damage. Renal microvascular endothelial cells are renowned Stx targets, whereas almost nothing is known on the involvement of renal epithelial cells in Stx-mediated kidney injury. In this study, we report for the first time on the identification and precise structural characterization of Stx-binding glycolipids in two porcine kidney-derived epithelial cell lines. Of note, one cell line was highly sensitive towards the cytotoxic action of Stx2e, whereas the other was refractory. The cell-damaging activity of Stx2e could be inhibited in the case of the toxin-sensitive cell line using synthesized neoglycolipids applied as neoglycolipid-spiked glycovesicles. Neoglycolipids, that resemble the native glycolipid receptors being exposed on the cell surface, compete with the genuine glycolipid receptors for binding to the toxin. When co-applied to in vitro Stx2e-exposed cells, neoglycolipids were capable to inhibit the cytotoxic activity of Stx2e. From our results we can conclude that the renal epithelial cells might be involved in Stx2e-mediated damage of the kidney, and thus in the manifestation of the edema disease in pigs. Future perspectives are the detection of the precise mechanisms of the cell surface interaction between toxin and glycolipid receptor, as well as the determination of the intracellular route of the toxin to its subcellular targets, where the toxin exerts its cytotoxic action.

## Figures and Tables

**Table 1:** Single nucleotide polymorphisms and amino acid exchanges of Stx subtypes used in this study.

Strain ^1^	Stx Subtype	Reference Sequence (GenBank acc. no.)	SNP Position (Nucleotide Exchange) ^2^	AA Codon Position (AA Exchange) ^3^
2074/97	Stx1a	*stx1a* (M19473)	200 (T → A)	67 (Thr → Ser)
03-06016	Stx2a	*stx2a* (EF441599)	170 (C → T)	57 (Leu → Ser)
2771/97	Stx2e(h)	*stx2e* (FM998846)	380 (T → C)	127 (Thr → Ile)
			887 (C → A)	296 (Thr → Lys)
S115G	Stx2e(p)	*stx2e* (FM998846)	33 (C → T)	no AA exchange;
			380 (T → C)	127 (Thr → Ile)
			937 (C → T)	313 (Pro → Ser)

^1^ Institutional numbering; ^2^ SNP, single nucleotide polymorphism; and ^3^ AA, amino acid.

## Supplementary Materials

The following are available online at https://www.mdpi.com/2076-2607/7/11/582/s1, Figure S1: Light microscopy micrographs of LLC-PK1 (**A**) and PK-15 cells (**B**), Figure S2: Orcinol stain (**A**) and antibody-mediated immunochemical detection of TLC-separated globo-series GSLs Gb3Cer (**B**) and Gb4Cer (**C**) in the neutral GSL preparations of the second biological replicate of porcine LLC-PK1 and PK-15 renal epithelial cell line, Figure S3: Detection of Stx-binding GSLs in the neutral GSL preparations of the second biological replicate of the porcine LLC-PK1 and PK-15 renal epithelial cell line, Table S1: Synopsis of *m/z* values and proposed structures of Stx GSL receptors Gb3Cer and Gb4Cer isolated from porcine LLC-PK1 renal epithelial cells, Table S2: Synopsis of *m/z* values and proposed structures of Stx GSL receptors Gb3Cer and Gb4Cer isolated from porcine PK-15 renal epithelial cells, Table S3: Synopsis of *m/z* values and proposed structures of Stx GSL receptor Gal_2_Cer species isolated from porcine PK-15 renal epithelial cells.

## References

[1] Imberechts H., De Greve H., Lintermans P. (1992). The pathogenesis of edema disease in pigs. A review. Vet. Microbiol..

[2] Van Beers-Schreurs H.M., Vellenga L., Wensing T., Breukin H.J. (1992). The pathogenesis of the post-weaning syndrome in weaned piglets: A review. Vet. Q..

[3] Moxley R.A. (2000). Edema disease. Vet. Clin. N. Am. Food Anim. Pract..

[4] Casanova N.A., Redondo L.M., Dailoff G.C., Arenas D., Fernández Miyakawa M.E. (2018). Overview of the role of Shiga toxins in porcine edema disease pathogenesis. Toxicon.

[5] Matise I., Sirinarumitr T., Bosworth B.T., Moon H.W. (2000). Vascular ultrastructure and DNA fragmentation in swine infected with Shiga toxin-producing *Escherichia coli*. Vet. Pathol..

[6] Benson K., Cramer S., Galla H.J. (2013). Impedance-based monitoring: Barrier properties and beyond. Fluids Barriers CNS.

[7] Meisen I., Rosenbrück R., Galla H.J., Hüwel S., Kouzel I.U., Mormann M., Karch H., Müthing J. (2013). Expression of Shiga toxin 2e glycosphingolipid receptors of primary porcine brain endothelial cells and toxin-mediated breakdown of the blood-brain barrier. Glycobiology.

[8] Moxley R.A., Duhamel G.E. (1999). Comparative pathology of bacterial enteric diseases of swine. Adv. Exp. Med. Biol..

[9] Friedrich A.W., Bielaszewska M., Zhang W.L., Pulz M., Kuczius T., Ammon A., Karch H. (2002). *Escherichia coli* harboring Shiga toxin 2 gene variants: Frequency and association with clinical symptoms. J. Infect. Dis..

[10] Beutin L., Krause G., Zimmermann S., Kaulfuss S., Gleier K. (2004). Characterization of Shiga toxin-producing *Escherichia coli* strains isolated from human patients in Germany over a 3-year period. J. Clin. Microbiol..

[11] Sonntag A.K., Bielaszewska M., Mellmann A., Dierksen N., Schierack P., Wieler L.H., Schmidt M.A., Karch H. (2005). Shiga toxin 2e-producing *Escherichia coli* isolates from humans and pigs differ in their virulence profiles and interactions with intestinal epithelial cells. Appl. Environ. Microbiol..

[12] Thomas A., Cheasty T., Chart H., Rowe B. (1994). Isolation of Vero cytotoxin-producing *Escherichia coli* serotypes O9ab:H- and O101:H- carrying VT2 variant gene sequences from a patient with haemolytic uraemic syndrome. Eur. J. Clin. Microbiol. Infect. Dis..

[13] Fasel D., Mellmann A., Cernela N., Hächler H., Fruth A., Khanna N., Egli A., Beckmann C., Hirsch H.H., Goldenberger D. (2014). Hemolytic uremic syndrome in a 65-year-old male linked to a very unusual type of *stx2e*- and *eae*-harboring O51:H49 Shiga toxin-producing *Escherichia coli*. J. Clin. Microbiol..

[14] Wieler L.H., Bauerfeind R. (2003). STEC as a veterinary problem. Diagnostics and prophylaxis in animals. Methods Mol. Med..

[15] Zweifel C., Schumacher S., Beutin L., Blanco J., Stephan R. (2006). Virulence profiles of Shiga toxin 2e-producing *Escherichia coli* isolated from healthy pig at slaughter. Vet. Microbiol..

[16] Tseng M., Fratamico P.M., Manning S.D., Funk J.A. (2014). Shiga toxin-producing *Escherichia coli* in swine: The public health perspective. Anim. Health Res. Rev..

[17] Tseng M., Fratamico P.M., Bagi L., Manzinger D., Funk J.A. (2015). Shiga toxin-producing *E. coli* (STEC) in swine: Prevalence over the finishing period and characteristics of the STEC isolates. Epidemiol. Infect..

[18] Ercoli L., Farneti S., Ranucci D., Scuota S., Branclari R. (2015). Role of verocytotoxigenic *Escherichia coli* in the swine production chain. Int. J. Food Saf..

[19] Bitzan M., Klemt M., Steffens R., Müller-Wiefel D.E. (1993). Differences in verotoxin neutralizing activity of therapeutic immunoglobulins and sera from healthy controls. Infection.

[20] Gannon V.P., Gyles C.L. (1990). Characteristics of the Shiga-like toxin produced by *Escherichia coli* associated with porcine edema disease. Vet. Microbiol..

[21] Franke S., Gunzer F., Wieler L.H., Baljer G., Karch H. (1995). Construction of recombinant Shiga-like toxin IIv (SLT-IIv) and ist use in monitoring the SLT-IIv antibody status of pigs. Vet. Microbiol..

[22] Franke S., Harmsen D., Caprioli A., Pierard D., Wieler L.H., Karch H. (1995). Clonal relatedness of Shiga-like toxin-producing *Escherichia coli* O101 strains of human and porcine origin. J. Clin. Microbiol..

[23] Boyd B., Tyrrell G., Maloney M., Gyles C., Brunton J., Lingwood C. (1993). Alteration of the glycolipid binding specificity of the pig edema toxin from globotetraosyl to globotriaosyl ceramide alters in vivo tissue targeting and results in verotoxin 1-like disease in pigs. J. Exp. Med..

[24] Waddell T.E., Lingwood C.A., Gyles C.L. (1996). Interaction of verotoxin 2e with pig intestine. Infect. Immun..

[25] Waddell T.E., Coomber B.L., Gyles C.L. (1998). Localization of potential binding sites for the edema disease Verotoxin (VT2e) in pigs. Can. J. Vet. Res..

[26] Winter K.R.K., Stoffregen W.C., Dean-Nystrom E.A. (2004). Shiga toxin binding to isolated porcine tissues and peripheral blood leukocytes. Infect. Immun..

[27] Pohlenz J.F., Winter K.R., Dean-Nystrom E.A. (2005). Shiga-toxigenic *Escherichia coli*-inoculated neonatal piglets develop kidney lesions that are comparable to those in humans with hemolytic-uremic syndrome. Infect. Immun..

[28] DeGrandis S., Law H., Brunton J., Gyles C., Lingwood C.A. (1989). Globotetraosylceramide is recognized by the pig edema disease toxin. J. Biol. Chem..

[29] Keusch G.T., Jacewicz M., Acheson D.W.K., Donohue-Rolfe A., Kane A.V., McCluer R.H. (1995). Globotriaosylceramide, Gb3, is an alternative functional receptor for Shiga-like toxin 2e. Infect. Immun..

[30] Müthing J., Meisen I., Zhang W., Bielaszewska M., Mormann M., Bauerfeind R., Schmidt M.A., Friedrich A.W., Karch H. (2012). Promiscuous Shiga toxin 2e and its intimate relationship to Forssman. Glycobiology.

[31] Steil D., Bonse R., Meisen I., Pohlentz G., Vallejo G., Karch H., Müthing J. (2016). A topographical atlas of Shiga toxin 2e receptor distribution in the tissues of weaned piglets. Toxins.

[32] Steil D., Schepers C.L., Pohlentz G., Legros N., Runde J., Humpf H.U., Karch H., Müthing J. (2015). Shiga toxin glycosphingolipid receptors of Vero-B4 kidney epithelial cells and their membrane microdomain lipid environment. J. Lipid Res..

[33] Scheutz F., Teel L.D., Beutin L., Piérard D., Buvens G., Karch H., Mellmann A., Caprioli A., Tozzoli R., Morabito S. (2012). Multicenter evaluation of a sequence-based protocol for subtyping Shiga toxins and standardizing Stx nomenclature. J. Clin. Microbiol..

[34] Nakao H., Takeda T. (2000). *Escherichia coli* Shiga toxin. J. Nat. Toxins.

[35] Ray P.E., Liu X.H. (2001). Pathogenesis of Shiga toxin-induced hemolytic uremic syndrome. Pediatr. Nephrol..

[36] Bielaszewska M., Karch H. (2005). Consequences of enterohaemorrhagic *Escherichia coli* infection for the vascular endothelium. Thromb. Hemost..

[37] Müthing J., Schweppe C.H., Karch H., Friedrich A.W. (2009). Shiga toxins, glycosphingolipid diversity, and endothelial cell injury. Thromb. Haemost..

[38] Zoja C., Buelli S., Morigi M. (2010). Shiga toxin-associated hemolytic uremic syndrome: Pathophysiology of endothelial dysfunction. Pediatr. Nephrol..

[39] Melton-Celsa A., Mohawk K., Teel L., O’Brien A. (2012). Pathogenesis of Shiga-toxin producing *Escherichia coli*. Curr. Top. Microbiol. Immunol..

[40] Bauwens A., Betz J., Meisen I., Kemper B., Karch H., Müthing J. (2013). Facing glycosphingolipid-Shiga toxin interaction: Dire straits for endothelial cells of the human vasculature. Cell. Mol. Life Sci..

[41] Kiyokawa N., Taguchi T., Mori T., Uchida H., Sato N., Takeda T., Fujimoto J. (1998). Induction of apoptosis in normal human renal tubular epithelial cells by *Escherichia coli* Shiga toxins 1 and 2. J. Infect. Dis..

[42] Karpman D., Håkansson A., Perez M.T., Isaksson C., Carlemalm E., Caprioli A., Svanborg C. (1998). Apoptosis of renal cortical cells in the hemolytic-uremic syndrome: In vivo and in vitro studies. Infect. Immun..

[43] Kodama T., Nagayama K., Yamada K., Ohba Y., Akeda Y., Honda T. (1999). Induction of apoptosis in human renal proximal tubular epithelial cells by *Escherichia coli* verocytotoxin 1 in vitro. Med. Microbiol. Immunol..

[44] Hughes A.K., Stricklett P.K., Schmid D., Kohan D.E. (2000). Cytotoxic effect of Shiga toxin-1 on human glomerular epithelial cells. Kidney Int..

[45] Kaneko K., Kiyokawa N., Ohtomo Y., Nagaoka R., Yamashiro Y., Taguchi T., Mori T., Fujimoto J., Takeda T. (2001). Apoptosis of renal tubular cells in Shiga toxin-mediated hemolytic uremic syndrome. Nephron.

[46] Creydt V.P., Silberstein C., Zotta E., Ibarra C. (2006). Cytotoxic effect of Shiga toxin-2 holotoxin and its B subunit on human renal tubular epithelial cells. Microbes Infect..

[47] Silberstein C., Pistone Creydt V., Gerhardt E., Núñez P., Ibarra C. (2008). Inhibition of water absorption in human proximal tubular epithelial cells in response to Shiga toxin-2. Pediatr. Nephrol..

[48] Márquez L.B., Araoz A., Repetto H.A., Ibarra F.R., Silberstein C. (2016). Effects of shiga toxin 2 on cellular regeneration mechanisms in primary and three-dimensional cultures of human renal tubular epithelial cells. Microb. Pathog..

[49] Taguchi T., Uchida H., Kiyokawa N., Mori T., Sato N., Horie H., Takeda T., Fujimoto J. (1998). Verotoxins induce apoptosis in human renal tubular epithelium derived cells. Kidney Int..

[50] Bitzan M., Bickford B.B., Foster G.H. (2004). Verotoxin (Shiga toxin) sensitizes renal epithelial cells to increased heme toxicity: Possible implications for the hemolytic uremic syndrome. J. Am. Soc. Nephrol..

[51] Sood A., Mathew R., Trachtman H. (2001). Cytoprotective effect of curcumin in human proximal tubule epithelial cells exposed to Shiga toxin. Biochem. Biophys. Res. Commun..

[52] Nestoridi E., Kushak R.I., Duguerre D., Grabowski E.F., Ingelfinger J.R. (2005). Up-regulation of tissue factor activity on human proximal tubular epithelial cells in response to Shiga toxin. Kidney Int..

[53] Wilson C., Foster G.H., Bitzan M. (2005). Silencing of Bak ameliorates apoptosis of human proximal tubular epithelial cells by *Escherichia coli-derived* Shiga toxin 2. Infection.

[54] Lentz E.K., Leyva-Illades D., Lee M.S., Cherla R.P., Tesh V.L. (2011). Differential response of the human renal proximal tubular epithelial cell line HK-2 to Shiga toxin types 1 and 2. Infect. Immun..

[55] Psotka M.A., Obata F., Kolling G.L., Gross L.K., Saleem M.A., Satchell S.C., Mathieson P.W., Obrig T.G. (2009). Shiga toxin 2 targets the murine renal collecting duct epithelium. Infect. Immun..

[56] Rasooly R., Do P.M., Griffey S.M., Vilches-Moure J.G., Friedman M. (2010). Ingested Shiga toxin 2 (Stx2) causes histopathological changes in kidney, spleen, and thymus tissues and mortality in mice. J. Agric. Food Chem..

[57] Morace I., Pilz R., Federico G., Jennemann R., Krunic D., Nordström V., von Gerichten J., Marsching C., Schießl I.M., Müthing J. (2019). Renal globotriaosylceramide facilitates tubular albumin absorption and its inhibition protects against acute kidney injury. Kidney Int..

[58] Porubsky S., Federico G., Müthing J., Jennemann R., Gretz N., Büttner S., Obermüller N., Jung O., Hauser I.A., Gröne E. (2014). Direct acute tubular damage contributes to Shigatoxin-mediated kidney failure. J. Pathol..

[59] Handler J.S., Perkins F.M., Johnson J.P. (1980). Studies of renal cell function using cell culture techniques. Am. J. Physiol..

[60] Toutain H., Morin J.P. (1992). Renal proximal tubule cell cultures for studying drug-induced nephrotoxicity and modulation of phenotype expression by medium components. Ren. Fail..

[61] Sun M., Liu X., Cao S., He Q., Zhou R., Ye J., Li Y., Chen H. (2007). Inhibition of porcine circovirus type 1 and type 2 production in PK-15 cells by small interfering RNAs targeting the Rep gene. Vet. Microbiol..

[62] Cao L., Chen J., Wei Y., Shi H., Zhang X., Yuan J., Shi D., Liu J., Zhu X., Wang X. (2017). Porcine parvovirus induces activation of NF-κB signaling pathways in PK-15 cells mediated by toll-like receptors. Mol. Immunol..

[63] Huang B., Li J., Zhang X., Zhao Q., Lu M., Lv Y. (2017). RIG-1 and MDA-5 signaling pathways contribute to IFN-β production and viral replication in porcine circovirus virus type 2-infected PK-15 cells in vitro. Vet. Microbiol..

[64] Wang Z.J., Xu C.M., Song Z.B., Wang M., Liu Q.Y., Jiang P., Li Y.F., Bai J., Wang X.W. (2018). Vimentin modulates infectious porcine circovirus type 2 in PK-15 cells. Virus Res..

[65] Hull R.N., Cherry W.R., Weaver G.W. (1976). The origin and characteristics of a pig kidney cell strain, LLC-PK. In Vitro.

[66] Perantoni A., Berman J.J. (1979). Properties of Wilms’ tumor line (TuWi) and pig kidney line (LLC-PK1) typical of normal kidney tubular epithelium. In Vitro.

[67] Kinoshita Y., Fukase M., Takenaka M., Nakada M., Miyauchi A., Fujita T. (1985). Calcitonin-responsive clonal cell line from porcine kidney (PK (15)) in serum-free medium. Endocrinol. Jpn..

[68] Newman J.T., Smith K.O. (1972). Characteristics of a swine papovavirus. Infect. Immun..

[69] Dulac G.C., Afshar A. (1989). Porcine circovirus antigens in PK-15 cell line (ATCC CCL-33) and evidence of antibodies to circovirus in Canadian pigs. Can. J. Vet. Res..

[70] Kouzel I.U., Pohlentz G., Schmitz J.S., Steil D., Humpf H.U., Karch H., Müthing J. (2017). Shiga toxin glycosphingolipid receptors in human Caco-2 and HCT-8 colon epithelial cell lines. Toxins.

[71] Betz J., Bielaszewska M., Thies A., Humpf H.U., Dreisewerd K., Karch H., Kim K.S., Friedrich A.W., Müthing J. (2011). Shiga toxin glycosphingolipid receptors in microvascular and macrovascular endothelial cells: Differential association with membrane lipid raft microdomains. J. Lipid Res..

[72] Pohlentz G., Steil D., Rubin D., Mellmann A., Karch H., Müthing J. (2019). Pectin-derived neoglycolipids: Tools for differentiation of Shiga toxin subtypes and inhibitors of Shiga toxin-mediated cellular injury. Carbohydr. Polym..

[73] Steil D., Pohlentz G., Legros N., Mormann M., Mellmann A., Karch H., Müthing J. (2018). Combining mass spectrometry, surface acoustic wave interaction analysis, and cell viability assays for characterization of Shiga toxin subtypes of pathogenic *Escherichia coli* bacteria. Anal. Chem..

[74] Legros N., Dusny S., Humpf H.U., Pohlentz G., Karch H., Müthing J. (2017). Shiga toxin glycosphingolipid receptors and their lipid membrane ensemble in primary human blood-brain-barrier endothelial cells. Glycobiology.

[75] Müthing J., Egge H., Kniep B., Mühlradt P.F. (1987). Structural characterization of gangliosides from murine T lymphocytes. Eur. J. Biochem..

[76] Mellmann A., Bletz S., Böking T., Kipp F., Becker K., Schultes A., Prior K., Harmsen D. (2016). Real-time genome sequencing of resistant bacteria provides precision infection control in an institutional setting. J. Clin. Microbiol..

[77] Legros N., Pohlentz G., Steil D., Kouzel I.U., Liashkovich I., Mellmann A., Karch H., Müthing J. (2018). Membrane assembly of Shiga toxin glycosphingolipid receptors and toxin refractiveness of MDCK II epithelial cells. J. Lipid Res..

[78] Meisen I., Friedrich A.W., Karch H., Witting U., Peter-Katalinić J., Müthing J. (2005). Application of combined high-performance thin-layer chromatography immunostaining and nanoESI-QTOF tandem mass spectrometry to the full structural characterization of high- and low-affinity binding ligands of Shiga toxin 1. Rapid Commun. Mass Spectrom..

[79] Legros N., Ptascheck S., Pohlentz G., Karch H., Dobrindt U., Müthing J. (2019). PapG subtype-specific binding characteristics of *Escherichia coli* towards globo-series glycosphingolipids of human kidney and bladder uroepithelial cells. Glycobiology.

[80] Legros N., Pohlentz G., Runde J., Dusny S., Humpf H.U., Karch H., Müthing J. (2017). Colocalization of receptors for Shiga toxins with *lipid rafts* in primary human renal glomerular endothelial cells and influence of D-PDMP on synthesis and distribution of glycosphingolipid receptors. Glycobiology.

[81] Schweppe C.H., Hoffmann P., Nofer J.R., Pohlentz G., Mormann M., Karch H., Friedrich A.W., Müthing J. (2010). Neutral glycosphingolipids in human blood: A precise mass spectrometry analysis with special reference to lipoprotein-associated Shiga toxin receptors. J. Lipid Res..

[82] Domon B., Costello C.E. (1988). A systematic nomenclature for carbohydrate fragmentations in FAB-MS/MS spectra of glycoconjugates. Glycoconj. J..

[83] Domon B., Costello C.E. (1988). Structure elucidation of glycosphingolipids and gangliosides using high-performance tandem mass spectrometry. Biochemistry.

[84] Müthing J., Distler U. (2010). Advances on the compositional analysis of glycosphingolipids combining thin-layer chromatography with mass spectrometry. Mass Spectrom. Rev..

[85] Meisen I., Mormann M., Müthing J. (2011). Thin-layer chromatography, overlay technique and mass spectrometry: A versatile triad advancing glycosphingolipidomics. Biochim. Biophys. Acta.

[86] Hsu F.F., Turk J., Stewart M.E., Downing D.T. (2002). Structural studies on ceramides as lithiated adducts by low energy collisional-activated dissociation tandem mass spectrometry with electrospray ionization. J. Am. Soc. Mass Spectrom..

[87] Sonnino S., Prinetti A. (2013). Membrane domains and the “lipid raft” concept. Curr. Med. Chem..

[88] Lingwood C.A., Binnington B., Manis A., Branch D.R. (2010). Globotriaosyl ceramide receptor function—Where membrane structure and pathology intersect. FEBS Lett..

[89] Sandvig K., Bergan J., Kavaliauskiene S., Skotland T. (2014). Lipid requirement for entry of protein toxins into cells. Prog. Lipid Res..

[90] Aigal S., Claudinon J., Römer W. (2015). Plasma membrane reorganization: A glycolipid gateway for microbes. Biochim. Biophys. Acta.

[91] Khan F., Proulx F., Lingwood C.A. (2009). Detergent-resistant globotriaosyl ceramide may define verotoxin/glomeruli-restricted hemolytic uremic syndrome. Kidney Int..

[92] Ray P.E. (2009). Shiga-like toxins and HIV-1 ‘go through’ glycosphingolipids and lipid rafts in renal cells. Kidney Int..

[93] Brown D.A. (2006). Lipid rafts, detergent-resistant membranes, and raft targeting signals. Physiology.

[94] Lingwood D., Simons K. (2007). Detergent resistance as a tool in membrane research. Nat. Protoc..

[95] Morris R.J., Jen A., Warley A. (2011). Isolation of nano-meso scale detergent resistant membrane that has properties expected of lipid ‘rafts’. J. Neurochem..

[96] Riske K.A., Domingues C.C., Casadei B.R., Mattei B., Caritá A.C., Lira R.B., Preté P.S.C., de Paula E. (2017). Biophysical approaches in the study of membrane solubilization: Quantitative assessment and the role of lateral inhomogeneity. Biophys. Rev..

[97] Legros N., Pohlentz G., Steil D., Müthing J. (2018). Shiga toxin-glycosphingolipid interaction: Status quo of research with focus on primary human brain and kidney endothelial cells. Int. J. Med. Microbiol..

[98] Johannes L. (2017). Shiga toxin—A model for glycolipid-dependent and lectin-driven endocytosis. Toxins.

[99] Sandvig K., Garred O., Prydz K., Kozlov J.V., Hansen S.H., van Deurs B. (1992). Retrograde transport of endocytosed Shiga toxin to the endoplasmic reticulum. Nature.

[100] Sandvig K., Kavaliauskiene S., Skotland T. (2018). Clathrin-independent endocytosis: An increasing degree of complexity. Histochem. Cell Biol..

[101] Endo Y., Tsurugi K., Yutsudo T., Takeda Y., Ogasawara T., Igarashi K. (1988). Site of action of a Vero toxin (VT2) from *Escherichia coli* O157:H7 and of Shiga toxin on eukaryotic ribosomes. RNA *N*-glycosidase activity of the toxins. Eur. J. Biochem..

[102] Brigotti M., Accorsi P., Carnicelli D., Rizzi S., González Vara A., Montanaro L., Sperti S. (2001). Shiga toxin 1: Damage to DNA in vitro. Toxicon.

[103] Brigotti M., Alfieri R., Sestili P., Bonelli M., Petronini P.G., Guidarelli A., Barbieri L., Stirpe F., Sperti S. (2002). Damage to nuclear DNA induced by Shiga toxin 1 and ricin in human endothelial cells. FASEB J..

[104] Lee M.S., Koo S., Jeong D.G., Tesh V.L. (2016). Shiga toxins as multi-functional proteins: Induction of host cellular stress responses, role in pathogenesis and therapeutic applications. Toxins.

[105] Toval F., Schiller R., Meisen I., Putze J., Kouzel I.U., Zhang W., Karch H., Bielaszewska M., Mormann M., Müthing J. (2004). Characterization of urinary tract infection-associated Shiga toxin-producing *Escherichia coli*. Infect. Immun..

[106] Totsika M., Moriel D.G., Idris A., Rogers B.A., Wurpel D.J., Phan M.D., Paterson D.L., Schembri M.A. (2012). Uropathogenic *Escherichia coli* mediated urinary tract infection. Curr. Drug Targets.

[107] Terlizzi M.E., Gribaudo G., Maffei M.E. (2017). Uropathogenic *Escherichia coli* (UPEC) infections: Virulence, factors, bladder responses, antibiotic, and non-antibiotic antimicrobial strategies. Front. Microbiol..

[108] Westerlund-Wikström B., Korhonen T.K. (2005). Molecular structure of adhesin domains in *Escherichia coli* fimbriae. Int. J. Med. Microbiol..

[109] Lane M.C., Mobley H.L. (2007). Role of P-fimbrial-mediated adherence in pyelonephritis and persistence of uropathogenic *Escherichia coli* (UPEC) in the mammalian kidney. Kidney Int..

[110] Kamath V.P., Yeske R.E., Gregson J.M., Ratcliffe R.M., Fang Y.R., Palcic M.M. (2004). Large-scale chemical and chemo-enzymatic synthesis of a spacer-containing Pk-trisaccharide. Carbohydr. Res..

[111] Kulkarni A.A., Weiss A.A., Iyer S.S. (2010). Glycan-based high-affinity ligands for toxins and pathogen receptors. Med. Res. Rev..

[112] Melton-Celsa A.R., O’Brien A.D. (2014). New therapeutic developments against Shiga toxin-producing *Escherichia coli*. Microbiol. Spectr..

[113] MacConnachie A.A., Todd W.T. (2004). Potential therapeutic agents for the prevention and treatment of haemolytic uraemic syndrome in Shiga toxin producing *Escherichia coli* infection. Curr. Opin. Infect. Dis..

