# Supplementary material for: Structural Insights into Escherichia coli Shiga Toxin (Stx) Glycosphingolipid Receptors of Porcine Renal Epithelial Cells and Inhibition of Stx-Mediated Cellular Injury Using Neoglycolipid-Spiked Glycovesicles
Source: Microorganisms. 2019 Nov 19;7(11):582. doi: 10.3390/microorganisms7110582 (PMC6920957; doi:10.3390/microorganisms7110582)
Supplement: Supplementary file 1 [file microorganisms-07-00582-s001.pdf]

Supplementary Materials:

Structural Insights into *Escherichia coli* Shiga Toxin (Stx) Glycosphingolipid Receptors of Porcine Renal Epithelial Cells and Inhibition of Stx-Mediated Cellular Injury Using Neoglycolipid-Spiked Glycovesicles

Johanna Detzner [1], Caroline Gloerfeld [1], Gottfried Pohlentz [1], Nadine Legros [1], Hans-Ulrich Humpf [2], Alexander Mellmann [1], Helge Karch [1] and Johannes Müthing [1]

[1] Institute for Hygiene, University of Münster, D-48149 Münster, Germany

[2] Institute for Food Chemistry, University of Münster, D-48149 Münster, Germany

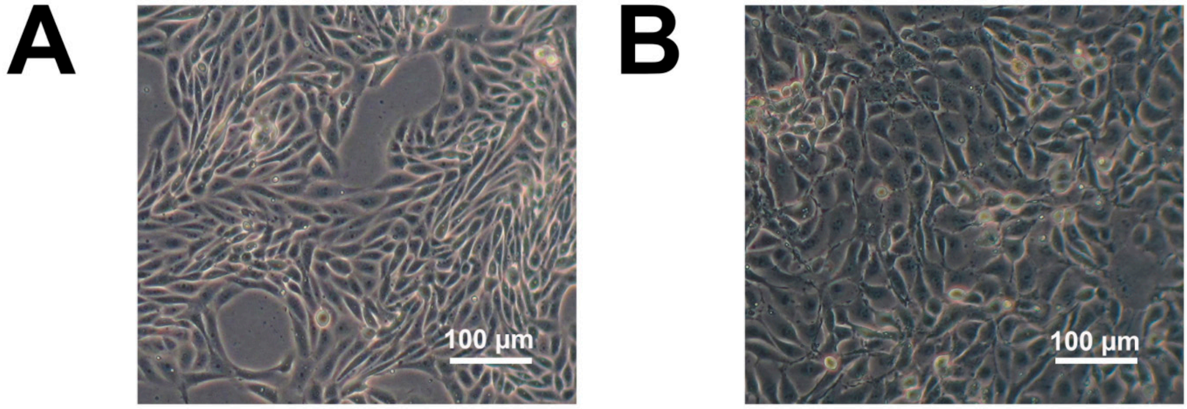

Figure S1. Light microscopy micrographs of LLC-PK1 (**A**) and PK-15 cells (**B**). Pictures were taken from cells of passage 12 at approximate 80% confluence of LLC-PK1 (**A**) and approximate 95% confluence of PK-15 cells (**B**). Original magnification x10.

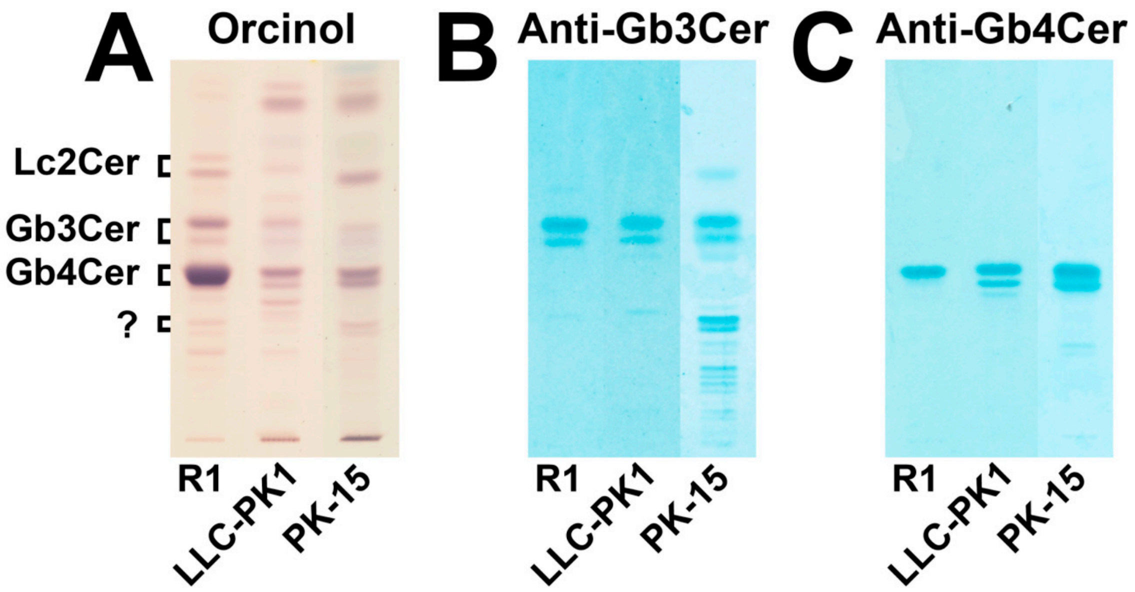

Figure S2. Orcinol stain (**A**) and antibody-mediated immunochemical detection of TLC-separated globo-series GSLs Gb3Cer (**B**) and Gb4Cer (**C**) in the neutral GSL preparations of the second biological replicate of the porcine LLC-PK1 and PK-15 renal epithelial cell line. The applied GSL quantities correspond to 2 x 10^6 LLC-PK1 and 1 x 10^6 PK-15 cells for the orcinol stain (**A**) and to 5 x 10^5 LLC-PK1 and PK-15 cells for the anti-Gb3Cer (**B**) and anti-Gb4Cer overlay assay (**C**). R1: 20 µg (**A**), 2 µg (**B**), and 0.2 µg (**C**) of neutral GSLs from human erythrocytes served as reference.

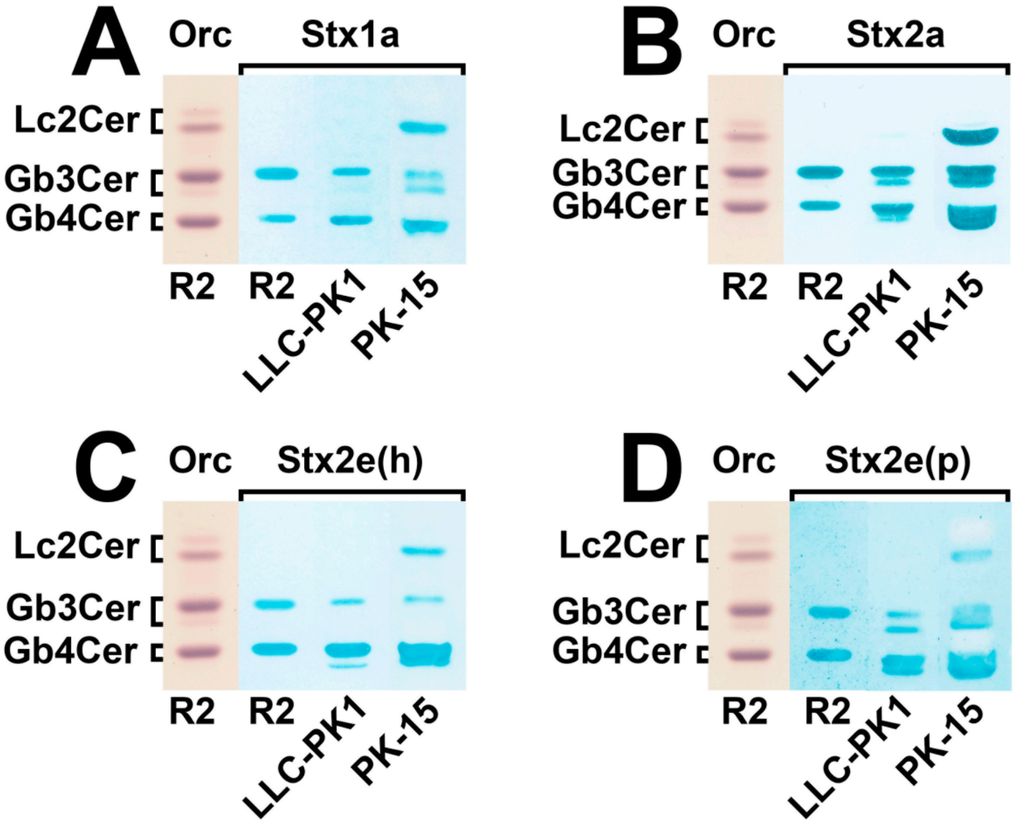

Figure S3. Detection of Stx-binding GSLs in the neutral GSL preparations of the second biological replicate of the porcine LLC-PK1 and PK-15 renal epithelial cell line. (**A**-**D**) Stx1a and Stx2a subtypes originated from human EHEC isolates. The two Stx2e variants are of different origin: Stx2e(h) derived from a human and Stx2(p) from a porcine STEC isolate. Applied GSL amounts correspond to 2 x 10^6 cells for the Stx overlay assays. R2: 20 µg and 2.4 µg of an equimolar mixture of Gb3Cer and Gb4Cer served as reference for the orcinol (Orc) stains and the Stx overlay assays, respectively.

Table S1. Synopsis of *m/z* values and proposed structures of Stx GSL receptors Gb3Cer and Gb4Cer isolated from porcine LLC-PK1 renal epithelial cells.[1]

m/z_{exp}[2]	m/z_{calc}[3]	GSL	Lipoform
**1046.66**	**1046.6603**	**Gb3Cer**	**d18:1, C16:0**
1062.65	1062.6552	Gb3Cer	d18:1, C16:0-OH
1128.73	1128.7386	Gb3Cer	d18:1, C22:1
**1130.75**	**1130.7542**	**Gb3Cer**	**d18:1, C22:0**
**1156.77**	**1156.7699**	**Gb3Cer**	**d18:1, C24:1**
**1158.79**	**1158.7855**	**Gb3Cer**	**d18:1, C24:0**
1172.77	1172.7648	Gb3Cer	d18:1, C24:1-OH
1174.77	1174.7803	Gb3Cer	d18:1, C24:0-OH
**1249.74**	**1249.7397**	**Gb4Cer**	**d18:1, C16:0**
1265.74	1265.7346	Gb4Cer	d18:1, C16:0-OH
1277.79	1277.7710	Gb4Cer	d18:1, C18:0
1305.81	1305.8023	Gb4Cer	d18:1, C20:0
1331.82	1331.8179	Gb4Cer	d18:1, C22:1
**1333.83**	**1333.8336**	**Gb4Cer**	**d18:1, C22:0**
**1359.85**	**1359.8492**	**Gb4Cer**	**d18:1, C24:1**
**1361.86**	**1361.8649**	**Gb4Cer**	**d18:1, C24:0**
1375.85	1375.8441	Gb4Cer	d18:1, C24:1-OH
1377.86	1377.8598	Gb4Cer	d18:1, C24:0-OH
1387.89	1387.8805	Gb4Cer	d18:1, C26:1

[1] Prevalent GSL species are highlighted in bold type (cf. Figure 3; [2] m/z_{exp}, experimental *m/z* values; [3] m/z_{calc}, calculated *m/z* values.

Table S2. Synopsis of *m*/*z* values and proposed structures of Stx GSL receptors Gb3Cer and Gb4Cer isolated from porcine PK-15 renal epithelial cells.[1]

m/z_{exp}[2]	m/z_{calc}[3]	GSL	Lipoform
1046.66	1046.6603	Gb3Cer	d18:1, C16:0
**1156.77**	**1156.7699**	**Gb3Cer**	**d18:1, C24:1**
**1158.79**	**1158.7855**	**Gb3Cer**	**d18:1, C24:0**
**1249.74**	**1249.7397**	**Gb4Cer**	**d18:1, C16:0**
1265.74	1265.7346	Gb4Cer	d18:1, C16:0-OH
**1277.79**	**1277.7710**	**Gb4Cer**	**d18:1, C18:0**
**1305.81**	**1305.8023**	**Gb4Cer**	**d18:1, C20:0**
**1331.82**	**1331.8179**	**Gb4Cer**	**d18:1, C22:1**
**1333.85**	**1333.8336**	**Gb4Cer**	**d18:1, C22:0**
1357.84	1357.8336	Gb4Cer	d18:1, C24:2
**1359.85**	**1359.8492**	**Gb4Cer**	**d18:1, C24:1**
**1361.86**	**1361.8649**	**Gb4Cer**	**d18:1, C24:0**
1373.84	1373.8285	Gb4Cer	d18:1, C24:2-OH
1375.85	1375.8441	Gb4Cer	d18:1, C24:1-OH
1377.86	1377.8598	Gb4Cer	d18:1, C24:0-OH

[1] Prevalent GSL species are highlighted in bold type (cf. Figure 4); [2] m/z_{exp}, experimental *m*/*z* values; [3] m/z_{calc}, calculated *m*/*z* values.

Table S3. Synopsis of *m/z* values and proposed structures of Stx GSL receptor Gal_2Cer species isolated from porcine PK-15 renal epithelial cells.[1.]

m/z_{exp}[2]	m/z_{calc}[3]	GSL	Lipoform
884.62	884.6075	Gal_2Cer	d18:1, C16:0
956.67	956.6650	Gal_2Cer	d18:1, C20:0-OH
**982.68**	**982.6807**	**Gal_2Cer**	**d18:1, C22:1-OH**
**984.69**	**984.6963**	**Gal_2Cer**	**d18:1, C22:0-OH**
996.70	996.6963	Gal_2Cer	d18:1, C23:1-OH
998.72	998.7120	Gal_2Cer	d18:1, C23:0-OH
**1008.70**	**1008.6963**	**Gal_2Cer**	**d18:1, C24:2-OH**
**1010.71**	**1010.7120**	**Gal_2Cer**	**d18:1, C24:1-OH**
**1012.72**	**1012.7276**	**Gal_2Cer**	**d18:1, C24:0-OH**
1036.73	1036.7276	Gal_2Cer	d18:1, C26:2-OH
1038.74	1038.7433	Gal_2Cer	d18:1, C26:1-OH

[1] Prevalent GSL species are highlighted in bold type (cf. Figure 5); [2] m/z_{exp}, experimental *m/z* values; [3] m/z_{calc}, calculated *m/z* values.
